# Atomoxetine Treatment Strengthens an Anti-Correlated Relationship between Functional Brain Networks in Medication-Naïve Adults with Attention-Deficit Hyperactivity Disorder: A Randomized Double-Blind Placebo-Controlled Clinical Trial

**DOI:** 10.1093/ijnp/pyv094

**Published:** 2015-09-16

**Authors:** Hsiang-Yuan Lin, Susan Shur-Fen Gau

**Affiliations:** Department of Psychiatry, National Taiwan University Hospital and College of Medicine, Taipei, Taiwan (Drs Lin and Gau);; Graduate Institute of Brain and Mind Sciences, and Graduate Institute of Clinical Medicine, National Taiwan University College of Medicine, Taipei, Taiwan (Dr Gau).

**Keywords:** attention-deficit hyperactivity disorder, atomoxetine, resting-state fMRI, randomized double-blind placebo-controlled clinical trial, adult

## Abstract

**Background:**

Although atomoxetine demonstrates efficacy in individuals with attention-deficit hyperactivity disorder, its treatment effects on brain resting-state functional connectivity remain unknown. Therefore, we aimed to investigate major brain functional networks in medication-naïve adults with attention-deficit hyperactivity disorder and the efficacy of atomoxetine treatment on resting-state functional connectivity.

**Methods:**

After collecting baseline resting-state functional MRI scans from 24 adults with attention-deficit hyperactivity disorder (aged 18–52 years) and 24 healthy controls (matched in demographic characteristics), the participants with attention-deficit hyperactivity disorder were randomly assigned to atomoxetine (n=12) and placebo (n=12) arms in an 8-week, double-blind, placebo-controlled trial. The primary outcome was functional connectivity assessed by a resting-state functional MRI. Seed-based functional connectivity was calculated and compared for the affective, attention, default, and cognitive control networks.

**Results:**

At baseline, we found atypical cross talk between the default, cognitive control, and dorsal attention networks and hypoconnectivity within the dorsal attention and default networks in adults with attention-deficit hyperactivity disorder. Our first-ever placebo-controlled clinical trial incorporating resting-state functional MRI showed that treatment with atomoxetine strengthened an anticorrelated relationship between the default and task-positive networks and modulated all major brain networks. The strengthened anticorrelations were associated with improving clinical symptoms in the atomoxetine-treated adults.

**Conclusions:**

Our results support the idea that atypical default mode network task-positive network interaction plays an important role in the pathophysiology of adult attention-deficit hyperactivity disorder. Strengthening this atypical relationship following atomoxetine treatment suggests an important pathway to treat attention-deficit hyperactivity disorder.

## Introduction

Attention-deficit hyperactivity disorder (ADHD) is an early-onset neurodevelopmental disorder with clinical symptoms often persisting into adulthood (Fayyad et al., 2007). Given considerable heterogeneity (Castellanos et al., 2006), neuroimaging studies may provide insights into the mechanism underpinning individuals who have persistent ADHD from childhood to adulthood.

Intrinsic resting-state functional connectivity (RSFC), represented by the correlation of low-frequency (eg, <0.1 Hz) spontaneous fluctuations in neural activity measured by a resting-state functional MRI (rs-fMRI) BOLD signal, can reliably characterize the functional organization of the brain at a systems level (Castellanos et al., 2013). Aberrant neural connectivity and synchrony across brain regions has emerged as a characteristic of brain differences in ADHD (Posner et al., 2014). Previous rs-fMRI studies reported reduced positive connections between the midline hub regions of the default mode network (DMN) (Fair et al., 2010) and a reduced anticorrelated relationship between the DMN and control network (Castellanos et al., 2008; Hoekzema et al., 2014) alongside dorsal attention network (Tomasi and Volkow, 2012) in ADHD. These 2 specific interactions in the DMN are dissociated in adults with and without persistent ADHD (Mattfeld et al., 2014). However, McCarthy and colleagues (2013) disparately found hyperconnectivity within the DMN, alongside increased RSFC in the affective and control networks, but hypoconnectivity within the attention networks in adults with ADHD. Sample heterogeneity in terms of psychotropic exposure might partially confound these findings (Qi et al., 2014), suggesting a pressing need for studies with a medication-naïve patient cohort (Hoekzema et al., 2014).

Pharmacological treatment is recommended as the first-line treatment for adults with ADHD (Seixas et al., 2012). Atomoxetine, the first approved nonstimulant ADHD treatment, has demonstrated its efficacy and tolerability for treating adults with ADHD (Ni et al., 2013a) and could improve their executive functions (Ni et al., 2013b). Atomoxetine is thought to mainly act at promiscuous presynaptic norepinephrine transporters (NETs) that clear both norepinephrine and dopamine in the prefrontal regions (Bymaster et al., 2002). Functional imaging studies generally suggest that positive responses to atomoxetine may reflect acute actions on the executive functions of the prefrontal cortex (Cubillo et al., 2014a, 2014b; Nandam et al., 2014). However, there are likely important psychopharmacologic differences between single-challenge doses of medication and treatment administered over a more extended period, particularly for atomoxetine, which takes weeks to exert its clinical effects (Newcorn et al., 2009).

Only 2 studies have investigated atomoxetine treatment effects on neural activity and its relationship with clinical improvement. After an 8-week treatment with atomoxetine, adults with ADHD had increased fronto-parietal activation during interference (Bush et al., 2013). In a 6- to 8-week comparison study, Schulz and colleagues (2012) found divergent associations with gains in inhibitory-related activation for atomoxetine and reductions in activation for methylphenidate in the right inferior frontal gyrus, left supplementary motor area, and bilateral posterior cingulate cortex (PCC). However, to our knowledge, there is currently no information about the changes in RSFC after atomoxetine treatment.

The aim of the present study was thus 2-fold. First, we used rs-fMRI to explore ADHD-related RSFC differences between medication-naïve adults with ADHD and healthy controls in the 5 predefined neural networks (ie, the DMN, affective, dorsal and ventral attention, and cognitive control networks; see supplementary Material for the rationales of selecting these networks). Second, we investigated how an 8-week treatment with atomoxetine affected RSFC in medication-naïve adults with ADHD. We further probed how posttreatment changes in RSFC were related to clinical and neuropsychological performances in the atomoxetine-treated group. Based on earlier studies, we hypothesized that medication-naïve adults with ADHD would exhibit reduced anticorrelated relationships between the DMN and task-positive networks (attention and control networks), as well as decreased RSFC in the DMN, compared with controls. Further decreased connections in the cognitive control and attention network and increased RSFC in the affective network would be found in the patient group relative to the control group. These changes might demonstrate some patterns distinct from the report of McCarthy and colleagues (2013) in a medicated patient cohort, while we did not hold specific hypotheses regarding the difference given that chronic effects of medication on RSFC in ADHD remain unknown. We also hypothesized that atomoxetine treatment would modulate RSFC in major brain networks, especially the task-positive networks with principal input from the prefrontal region. With chronic treatment of atomoxetine, RSFC within the networks would be increased and anticorrelations between the DMN and task-positive networks would be strengthened as clinical symptoms and neuropsychological performances improved.

## Methods

### Overall Study Design and Ethics

This study consisted of a case-control study to compare the functional connectivity between 24 medication-naïve adults with ADHD and 24 matched healthy controls, and an 8-week atomtoxetine treatment, double-blind, placebo-controlled clinical trial on these 24 adults with ADHD. The Research Ethics Committee at the National Taiwan University Hospital approved the study procedures (IRB ID: 200903059M; ClinicalTrials.gov no. NCT00917371). All participants provided written informed consent.

### Participants

All the 48 participants, aged 18 to 52 years old, were free of significant medical problems and received the same clinical, psychiatric, neuropsychological, and MRI assessments. The patients fulfilled *DSM-IV-TR* criteria for childhood and current ADHD diagnosed by the corresponding author and further confirmed with the semistructured Conners’ Adult ADHD Diagnostic Interview as described in the DSM-IV (Multi-Health Systems Inc.) (Conners et al., 1999) for current ADHD, and the modified adult version of the ADHD supplement of the Chinese version of the Schedule for Affective Disorders and Schizophrenia–Epidemiological Version for past and current ADHD (Ni et al., 2013a, 2013b). They were recruited at the Department of Psychiatry, National Taiwan University Hospital, Taipei, Taiwan.

Twenty-four healthy adult controls without any lifetime diagnosis of ADHD based on the same clinical and psychiatric assessments as the ADHD group were recruited from the community according to the same age, sex, intelligence, and handedness of the ADHD group.

All participants who had any systemic medical illness; a history of bipolar disorder, psychosis, major depression, substance use disorder, pervasive developmental disorder; or currently had depressive or anxiety symptoms or suicidal ideations; had been treated with any psychotropic agents, including medications for ADHD; or IQ <80 as assessed by the Wechsler Adult Intelligence Scale-Revised were excluded from this study.

### Procedure

All enrolled participants underwent clinical, neuropsychological, and imaging assessments. Control participants completed a single MRI scan and the Rapid Visual Information Processing (RVP) of the Cambridge Neuropsychological Test Automated Battery (Gau and Huang, 2014); adults with ADHD completed pre- and posttreatment MRI scans (for 8 weeks), the RVP task, and their reports on the Chinese version of the Adult ADHD Self-Report Scale (ASRS) (Yeh et al., 2008). The total hits of RVP were used to assess sustained attention and index attention capacity (Gau and Huang, 2014). The 18-item ASRS was used to assess adult ADHD symptoms of inattention (items 1–9) and hyperactivity/impulsivity (items 10–18) (Yeh et al., 2008). Full descriptions of the RVP and ASRS are provided in the supplementary Material.

**Figure 1.**
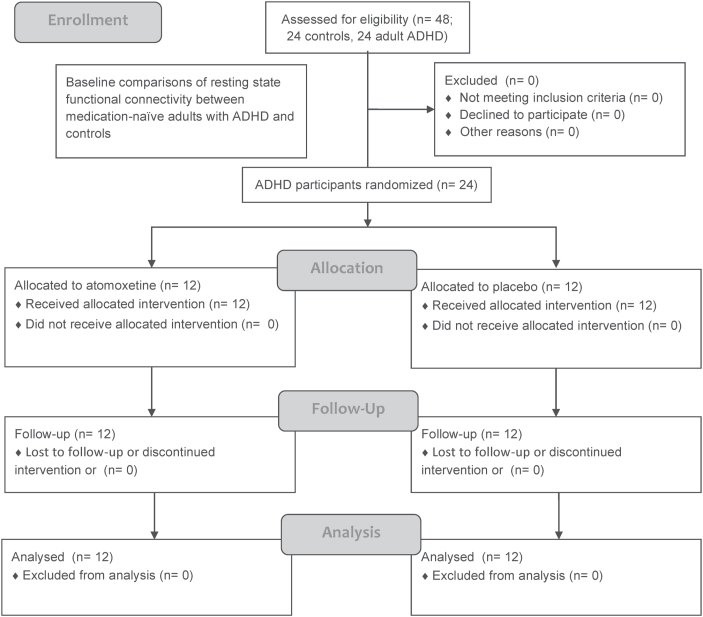
Flow diagram of the procedure of the clinical trial.

Medication-naïve adults with ADHD were randomly assigned to double-blind treatment with atomoxetine (n=12) or placebo (n=12) according to computer-generated random sequencing. Participants with ADHD were initially administered atomoxetine 0.5mg/kg/d in the morning at baseline and would titrate the drug dosage at week 2 (usually reaching the optimal dose, 1.2mg/kg/d), and at week 4 depending on clinical response and adverse effects (maximum daily dosage of atomoxetine=1.2mg/kg) depending on clinical response and adverse effects. Only nonspecific supportive psychotherapy and counseling were implemented as needed during the follow-up. All ADHD participants completed the pre- and posttreatment MRI scans and other required assessments without any missing data (Figure 1).

### MRI Acquisition and Preprocessing

Data were obtained on a 3T scanner (Siemens Magnetom Tim Trio) with a 32-channel phased-arrayed head coil. All participants were verbally instructed to remain still with their eyes closed to complete a 6-minute rs-fMRI scan (Van Dijk et al., 2010). Wakefulness was monitored and ensured at the end of scan by checking the participants’ prompt responses to technicians’ questions. All participants denied falling asleep during the scan. Standard functional imaging preprocessing (Yan and Zhang, 2010), combined with stringent motion artifacts correction procedure, and component-based (CompCor) approach for denoising (Behzadi et al., 2007; Whitfield-Gabrieli and Nieto-Castanon, 2012) were performed (see supplementary Material for imaging parameters and preprocessing details).

### Functional Connectivity Analysis

Following preprocessing, whole-brain functional connectivity was calculated by correlating the seed time-series with the time course of all other voxels using REST toolbox (Song et al., 2011). The resulting Pearson’s correlation coefficients were Fisher-z transformed to conform to normality assumptions for second-level analyses. To facilitate direct comparisons between the previous rs-fMRI findings in adults with ADHD and ours, a priori seeds with a 5-mm radius were defined following 2 previous rs-fMRI studies (McCarthy et al., 2013; Mattfeld et al., 2014) to explore connectivity within the DMN (seeded at the bilateral precuneus [PRE], PCC, and medial prefrontal cortex [mPFC]), affective (seeded in the bilateral subgenual anterior cingulate cortex [ACC]), dorsal (seeded at the bilateral frontal eye field, FEF, alongside inferior parietal sulcus, IPS) and ventral attention (seeded at the bilateral ventral frontal cortex, VFC, and temporoparietal junction [TPJ]), and cognitive control networks (seeded at the bilateral dorsolateral prefrontal cortex [DLPFC]) (seed coordinates in Table 1). We included findings of negative connectivity in the results, because the CompCor method is shown to allow for interpretations of anticorrelations (Chai et al., 2012).

**Table 1.** Coordinates of Seed Regions

Networks and seed regions	MNI Coordinates			Talairach Coordinates		
	x	y	z	x	y	z
Affective network						
Left subgenual ACC^*a*^	-10	39	-6	-10	35	2
Right subgenual ACC^*a*^	12	39	-6	10	35	2
Ventral attention network						
Left TPJ^*a*^	-56	-48	23	-53	-48	20
Right TPJ ^*a*^	59	-47	22	53	-48	20
Left VFC^*a*^	-39	21	-5	-37	18	1
Right VFC^*a*^	41	21	-6	37	18	1
Dorsal attention network						
Left IPS^*a*^	-27	-55	56	-27	-58	49
Right IPS^*a*^	31	-55	55	27	-58	49
Left FEF^*a*^	-24	-7	54	-24	-13	51
Right FEF^*a*^	28	-7	53	24	-13	51
Cognitive control network						
Left DLPFC^*a*^	-38	33	25	-36	27	29
Right DLPFC^*a*^	40	33	24	36	27	29
Default network						
Left PRE^*a*^	-6	-60	25	-7	-60	21
Right PRE^*a*^	9	-60	25	7	-60	21
PCC	15	-56	28	-	-	-
mPFC	-1	47	-4	-	-	-

Abbreviations: ACC, anterior cingulate cortex; DLPFC, dorsolateral prefrontal cortex; FEF, frontal eye field; IPS, inferior parietal sulcus; mPFC, medial prefrontal cortex; PCC, posterior cingulate cortex; PRE, precuneus; TPJ, temporoparietal junction; VFC, ventral frontal cortex.

^*a*^The coordinates conversion from Talairach space to MNI space was changed by the toolbox developed by Lancaster et al. (2007).

### Statistical Analysis

All behavioral data analysis was conducted using IBM SPSS Statistics for Macintosh (22.0, IBM Corp., Armonk, NY). The alpha value was preselected at *P*<.05. One-way ANOVA was used to determine differences in clinical symptoms, neuropsychological performances, vital signs, and measures of in-scanner head motion at baseline. One-way repeated-measures ANOVA was used to compare differences within subjects between weeks 8 to 10 and baseline in each treatment arm of the clinical trial. Mixed-effect ANOVA was used to test for treatment × time interaction effect.

To control the risks of false-positives, all significant clusters in neuroimaging-related statistical analyses were corrected for multiple comparisons at the cluster level by controlling topological Family-wise error (FWE) calculated based on random field theory implemented in SPM8, using a cluster-forming voxel-level height threshold of *P*<.01 and a spatial extent threshold (corrected for nonstationarity) that ensures a cluster-wise FWE at *P<*.05 (Hayasaka and Nichols, 2003; Hayasaka et al., 2004). xjView8 toolbox (http://www.alivelearn.net/xjview8/) was used to localize the significant clusters and the related Brodmann area (BA). The results were visualized using BrainNet Viewer (Xia et al., 2013).

For baseline comparisons, we analyzed rs-fMRI data in SPM8 using 2-sample *t* tests to determine significant differences in RSFC between ADHD and controls. For treatment effects, we entered each ADHD participant’s seed-based connectivity map into a 2×2 repeated-measure factorial model using SPM8. We treated time as a repeated measure with 2 levels: pretreatment and posttreatment scans. We used treatment as a between-group factor with 2 levels: atomoxetine and placebo. We isolated a time×treatment term to determine differential treatment effects on connectivity, followed by conducting a posthoc pairwise *t* test for seed-regions of interest connectivity to determine the nature of the interaction. As suggested by Yan and colleagues (2013), we included mean FD as a covariate in all group-level analyses to further account for motion artifacts. Nonetheless, because groups were matched on demographic variables, misuses of ANCOVA may lead to unpredictable results (Miller and Chapman, 2001; Suckling, 2011). There was limited sample size, and we did not include IQ and age as nuisance covariates in the models. This decision was justified by no correlation between the connectivity strengths of the identified clusters and these variables (supplementary Table 1).

The 2 treatment groups were comparable in demographic and clinical characteristics (Table 2). However, pretreatment connectivity differences between the atomoxetine and placebo arms could produce spurious interpretations of treatment × time interactions. To preclude this possibility, we compared the baseline scans in the atomoxetine vs placebo arms by conducting an *F* test on the functional connectivity maps for covarying mean FD, with an uncorrected *P<*.05. We then created a mask that excluded any voxels, in which there were baseline RSFC differences across the 2 treatment arms, from subsequent analyses. Owing to the finite spatial coverage of the EPI scan (102mm thickness), we further excluded the cerebellum in the analysis by subtracting the cerebellar regions derived from the Automated Anatomical Labeling template (Tzourio-Mazoyer et al., 2002) from the gray matter mask.

**Table 2.** Demographic and Clinical Characteristics of Study Participants

	**Control**	**Adult ADHD**			**Baseline Comparisons**	
		**All**	**Atomoxetine**	**Placebo**	**ADHD vs Control**	**Atomoxetine vs Placebo**
	(n=24)	(n=24)	(n=12)	(n=12)		
Male, n (%)	11 (46)	11 (46)	6 (50)	5 (41.67)	*χ* ^2^_(1)_=0.00, *P* =1.000	*χ* ^2^_(1)_=0.168, *P*=.682
Right handedness, n (%)	22 (92)	22 (92)	11 (92)	11 (92)	*χ* ^2^_(1)_=0.00, *P* =1.000	*χ* ^2^_(1)_=0.00, *P*=1.000
Age, mean (SD)	30.42 (8.95)	30.12 (9.15)	27.75 (8.17)	32.50 (9.80)	*F*_(1, 46)_=0.12, *P*=.912	*F*_(1, 22)_=1.66, *P*=.211
IQ						
Full-scale IQ	117.08 (10.59)	117.25 (13.67)	114.58 (13.56)	119.92 (13.83)	*F*_(1, 46)_=0.00, *P* =.963	*F*_(1, 22)_=0.91, *P*=.350
Performance IQ	117.71 (11.36)	116.29 (14.99)	110.92 (12.96)	121.67 (16.17)	*F*_(1, 46)_=0.14, *P* =.714	*F*_(1, 22)_=3.41, *P*=.078
Verbal IQ	114.29 (9.60)	116.13 (12.07)	115.58 (12.80)	116.67 (11.85)	*F*_(1, 46)_=0.34, *P*=.563	*F*_(1, 22)_=0.05, *P*=.832
Vital signs, mean (SD)						
Systolic pressure	…	119.08 (13.99)	116.42 (13.26)	120.58 (14.95)	…	*F*_(1, 22)_=0.26, *P*=.616
Diastolic pressure	…	77.44 (9.90)	76.75 (11.59)	77.58 (8.66)	…	*F*_(1, 22)_ *=*0.00, *P*=.946
Heart Rate	…	71.36 (10.45)	74.25 (10.63)	68.67 (10.39)	…	*F*_(1, 22)_=1.57, *P*=.207
Height (cm), mean (SD)	…	165.56 (8.25)	165.19 (6.96)	165.96 (9.75)	…	*F*_(1, 22)_=0.05, *P*=.822
Weight (kg), mean (SD)	…	67.19 (16.31)	69.85 (19.15)	64.31 (12.77)	…	*F*_(1, 22)_=0.71, *P*=.408
Clinical symptoms Adult Self-Report Scale, mean (SD)						
Inattention	9.60 (4.47)	27.04 (6.03)	26.42 (6.53)	27.67 (5.69)	*F*_(1, 46)_=135.96, *P* <.001	*F*_(1, 22)_=0.25, *P*=.622
Hyperactivity-impulsivity	5.52 (4.40)	19.92 (6.72)	19.17 (6.93)	20.67 (6.72)	*F*_(1, 46)_=83.76, *P* <.001	*F*_(1, 22)_=0.29, *P*=.596
Cambridge Neuropsychological Test Automated Battery Rapid Visual Information Processing						
Total hits	20.40 (4.33)	18.88 (3.32)	18.77 (2.55)	19.00 (4.11)	*F*_(1, 46)_=1.94, *P*=.230	*F*_(1, 22)_=0.03, *P*=.915

Abbreviation: ADHD, attention-deficit hyperactivity disorder.

Based on the published method (Sarpal et al., 2015), we subtracted the baseline scan from the follow-up scan (follow-up minus baseline) using ImCalc in SPM8 to correlate the changes in behavioral ratings with longitudinal changes in RSFC in the atomoxetine-treated group. The resulting image representing the change in correlation was then taken into a group-level multiple regression analysis with improvement in clinical symptoms and neuropsychological performances as a regressor, separately (ie, 3 measures, inattention and hyperactivity/impulsivity in the ASRS, alongside RVP total hits; per parameter established one multiple regression model). Because each participant had different levels of clinical symptoms and cognitive performances at baseline, improvement in behaviors was indexed by percentile scores with changes in the ASRS (baseline minus follow-up) and RVP total hits (follow-up minus baseline) divided by baseline ratings, respectively. This analysis was performed for each seed, explicitly masked within the binary, whole-brain cerebral gray matter mask. Data were extracted and plotted from average values of significant clusters identified.

## Results

At baseline, there were no group differences (ADHD vs control; atomoxetine vs placebo) in demographics, intelligence, or RVP performances except that adults with ADHD had higher clinical symptom severity compared with control participants (Table 2). After 8 weeks of treatment, we did not find any significant treatment × time interaction in symptom severity, physical evaluations, or neuropsychological performances. Using repeated-measures ANOVA measurements, ADHD participants treated with atomoxetine had significant symptomatic reductions in inattention (*F*_(1, 11)_=19.53, *P*=.001) and hyperactivity-impulsivity (*F*_(1, 11)_=15.01, *P*=.003) assessed by the Chinese ASRS and increased total correct hits in RVP (*F*_(1, 11)_=8.8, *P*=.013), while ADHD participants treated with the placebo had significant improvement only in inattention symptoms from baseline to week 8 (*F*_(1, 11)_=13.45, *P*=.004) (supplementary Table 2).

The 2 comparison groups (ADHD vs control; preatomoxetine vs preplacebo) were separately matched on the amount of composite movement in terms of mean FD, maximum FD, and number of outliers, in the baseline scans, except that the preplacebo group had higher mean maximum FD compared with the preatomoxetine group (*F*_(1, 10)_=4.52, *P*=.045). After treatment, the postplacebo group had higher mean FD compared with the postatomoxetine group (*F*_(1, 10)_=6.06, *P*=.022), while there was no significant difference between the postplacebo and postatomoxetine groups in maximum head displacement and jerky movement (supplementary Table 3; supplementary Figure 1).

### Baseline Scans

The spatial extents and main hubs of the 5 neural networks were identified using 1-sample *t* test for the control group as shown in supplementary Figure 2. The DMN consisted of the PCC/PRE, mPFC, angular gyrus, lateral temporal cortex, and hippocampus formation. The cognitive control network was composed of the DLPFC, inferior frontal gyrus, dorsal ACC, anterior insula, anterior inferior parietal lobule, and inferolateral temporal cortex. The dorsal attention network mainly involved the FEF and IPS. The ventral attention network included the TPJ, VFC, and supramarginal gyrus. The affective network included the subgenual ACC (Beckmann et al., 2009), amygdala, hypothalamus, anterior insula, hippocampus, and orbitofrontal cortex.

**Figure 2.**
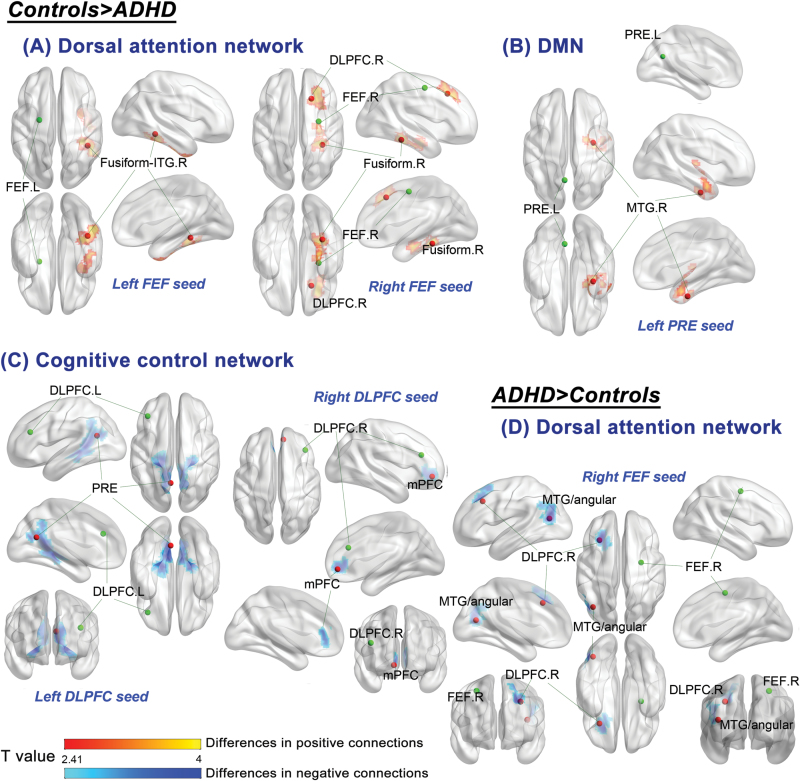
Differences in resting state functional connectivity of the major neural networks between controls and adults with attention-deficit hyperactivity disorder (ADHD) at baseline. Comparisons of the 2 groups demonstrated the controls had stronger positive connectivity in the (A) dorsal attention network between the left frontal eye field (FEF) and right fusiform/inferior temporal gyrus (ITG) and between the right FEF and right dorsolateral prefrontal cortex (DLPFC), and in the (B) default mode network (DMN) between the left precuneus (PRE) and right middle temporal gyrus (MTG). The control group also had greater negative connections in the (C) cognitive control network between the left DLPFC and PRE and between the right DLPFC and medial prefrontal cortex (mPFC). Adults with ADHD had stronger anticorrelations in the (D) dorsal attention network from the right FEF to left DLPFC and left MTG/angular gyrus. Statistical height threshold *P<*.01, FWE cluster-level corrected *P<*.05. The green dots represent the seed regions and the red dots indicate the regions showing atypical functional connectivity (peak coordinates). L, left; R, right.

For adults with ADHD, relative to the controls, weaker positive connections were found in the dorsal attention network, between the left FEF and right fusiform/inferior temporal gyrus (*P=*.014), between the right FEF and right parahippocampal gyurs/fusiform (*P=*.032), and between the right FEF and right middle frontal gyrus (BA 8, corresponding to the DLPFC; *P=*.015). The cognitive control network displayed reduced negative connectivity between the DLPFC and PRE/PCC for the ADHD group relative to the control group (*P<*.001). Adults with ADHD, compared with the controls, had weaker positive connections in the DMN between the left PRE and right middle temporal (MTG)/fusiform gyrus (*P=*.02). There was no hypoconnectivity in the affective network and ventral attention network for adults with ADHD compared with the controls (Figure 2; Table 3; supplementary Figure 2 for scatter plot of connectivity).

**Table 3.** Differences in Resting State Functional Connectivity between Controls and Adults with Attention-Deficit Hyperactivity Disorder (ADHD) at Baseline

**Network and Regions**	**MNI Coordinate**	**Cluster Size (voxels**)^*a*^	**Cluster-Level FWE- Corrected *P*** ^*b*^	**T Value**	**Functional Connectivity (Rz), Mean (SD**)	
					**Control**	**ADHD**
Control>ADHD						
Dorsal attention network						
Left FEF, right fusiform gyrus/inferior temporal gyrus (BA 20/37)	42, -42, -12	289	.014	T=5.1	0.23 (0.18)	-0.02 (0.14)
Right FEF, right parahippocampal/fusiform gyrus	33, -39, -18	252	.032	T=4.18	0.21 (0.15)	-0.01 (0.21)
Right FEF, right middle frontal gyrus (BA 8)	18, 24, 45	293	.015	T=4.71	0.17 (0.18)	-0.07 (0.21)
Cognitive control network						
Left DLPFC, precuneus/posterior cingulate gyrus (BA 31/30)^*c*^	-21, -36, -3	953	<.001	T=5.23	-0.29 (0.24)	-0.02 (0.20)
Right DLPFC, mPFC (BA 32/10)^*c*,*d*^	9, 48, -6	214	.053^*d*^	T=4.74	-0.15 (0.16)	0.05 (0.16)
DMN						
Left PRE, right middle temporal/fusiform gyrus (BA 20/21)	33, -9, -33	278	.02	T=4.59	0.29 (0.22)	-0.01 (0.17)
ADHD and Control with reversed patterns in terms of positive and negative connections						
Dorsal attention network						
Right FEF, left middle temporal/angular gyrus (BA 39)^*c*^	-39, -69, 15	277	.02	T=5.34	0.09 (0.17)	-0.15 (0.16)
Right FEF, left middle frontal gyrus (BA 8)^*c*^	-30, 24, 39	234	.045	T=4.71	0.08 (0.14)	-0.12 (0.18)

Abbreviations: BA, Brodmann area; DLPFC, dorsolateral prefrontal cortex; DMN, default mode network; FEF, frontal eye field; FWE, Family-wise error; MNI, Montreal Neurological Institute; mPFC, medial prefrontal cortex; PRE, precuneus; Rz, z-transformed Pearson’s correlational coefficient.

^*a*^The normalized voxel was resampled to the size of isotropic 3mm.

^*b*^The cluster-forming threshold was set at voxel-level *P<*.01.

^*c*^Differences were displayed in negative functional connectivity between the 2 groups.

^*d*^Trend-level significance.

In the dorsal attention network between the right FEF and left MTG/angular gyrus (BA 39; *P=*.02), alongside the left middle frontal gyrus (BA 8; *P=*.045), respectively, adults with ADHD displayed increased negative connections relative to the controls (ie, more propensity for negative connections in the pairs in the ADHD group, whereas positive connections prone in the control group). Adults with ADHD, relative to the controls, did not have significant hyperconnectivity in the cognitive control, ventral attention, affective, and DMNs (Figure 2; Table 3; supplementary Figure 3).

### Follow-Up Scans

**Figure 3.**
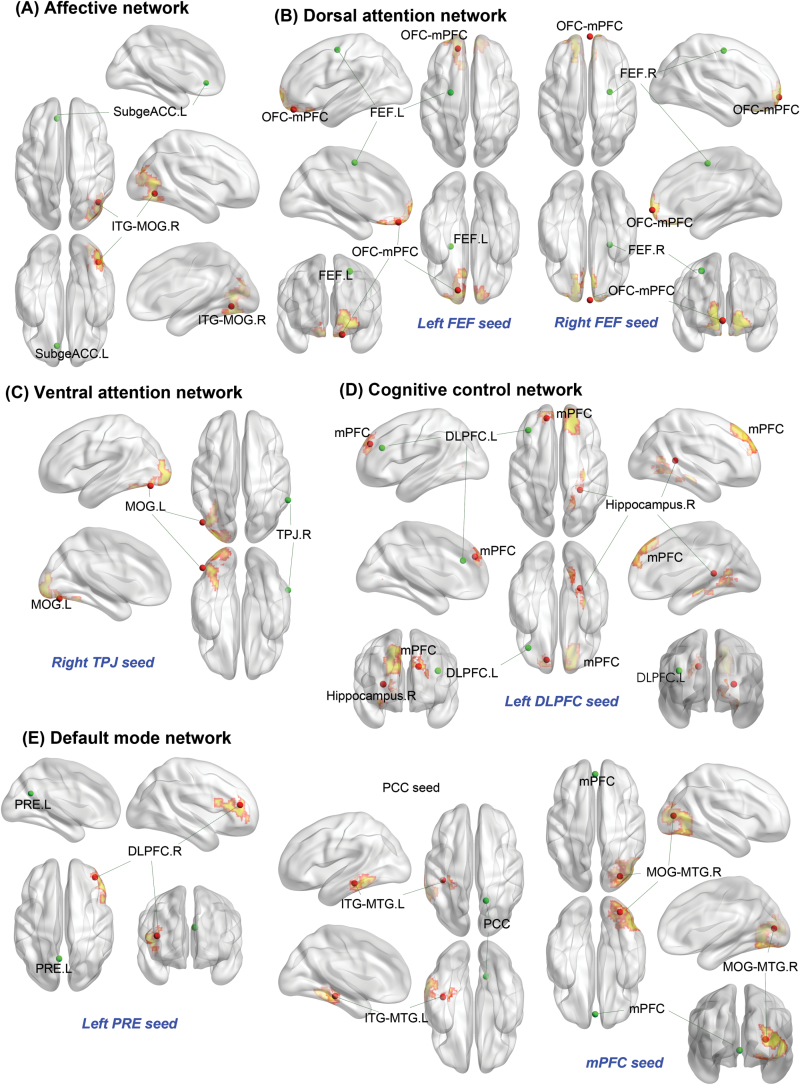
Connections demonstrating treatment × time interactions in the clinical trial. A mixed model for repeated measures revealed atomoxetine treatment modulated resting state functional connectivity across all the major neural networks investigated. Statistical height threshold *P<*.01, FWE cluster-level corrected *P<*.05. The green dots represent the seed regions and the red dots indicate the regions showing treatment × time interactions in the clinical trial (peak coordinates). The color (yellow areas with red edges) in the brain map displayed only the spatial extents of the clusters, but did not represent statistical values (see Table 4 for statistical values and functional connection strength). DLPFC,dorsolateral prefrontal cortex; FEF,frontal eye field; ITG,inferior temporal gyrus; L,left; MOG,middle occipital gyrus; mPFC,medial prefrontal cortex; MTG,middle temporal gyrus; OFC,orbitofrontal cortex; PCC,posterior cingulate cortex; PRE,precuneus; R,right; SubgeACC,subgenual anterior cingulate cortex; TPJ,temporoparietal junction.

Significant time × treatment interactions were extensively found throughout all major brain networks. After treatment with atomoxetine, we detected stronger negative connections in the cognitive control network between the left DLPFC and left superior frontal gyrus, medial part (corresponding to the mPFC; *P=*.006); in the DMN between the left PRE and right middle frontal gyrus, lateral part (corresponding to the DLPFC; *P=*.042), and between the PCC and left inferolateral temporal lobe (*P=*.024); and in the dorsal attention network between the bilateral FEF (*P=*.008) and orbitofrontal cortex/mPFC (*P=*.002), respectively. After treatment with atomoxetine, the posthoc analyses demonstrated increased connectivity strength in the DMN between the mPFC and right middle occipital/temporal gyrus (*P<*.001); in the affective network between the left subgenual ACC and right inferior temporal/middle occipital gyrus (*P=*.005); in the cognitive network between the left DLPFC and right hippocampus (*P=*.008); and in the right ventral attention network between the right TPJ and left middle occipital gyrus (*P*=.037) (Figure 3; Table 4; supplementary Figure 4).

**Table 4.** Connections Displaying Treatment × Time Interactions

Network and Regions	MNI Coordinate	Cluster Size (voxels)^*a*^	Cluster-level FWE- Corrected *P* ^*b*^	Interaction Term	Treatment Period	Connection Strength (Rz), Mean (SD)	
						Placebo	Atomoxetine
Affective network							
Left subgenual ACC, right inferior temporal/middle occipital gyrus (BA 37/19)	42, -66, -6	380	0.005	T=4.4	Post	-0.09 (0.13)	0.14 (0.15)
					Pre	0.05 (0.14)	-0.09 (0.19)
Right subgenual ACC	…						
Ventral attention network							
Left TPJ	…						
Right TPJ, left middle occipital gyrus (BA 18/19)	-48, -75, -15	253	0.037	T=3.97	Post	-0.16 (0.19)	0.08 (0.18)
					Pre	-0.10 (0.21)	-0.11 (0.16)
Left VFC	…						
Right VFC	…						
Dorsal attention network							
Left IPS	…						
Right IPS	…						
Left FEF, left orbitofrontal cortex/mPFC (BA 11/10)	-15, 48, -21	330	0.008	T=4.5	Post	0.07 (0.19)	-0.22 (0.18)
					Pre	-0.17 (0.16)	-0.04 (0.17)
Right FEF, orbitofrontal cortex/mPFC (BA 11/10)	3, 63, -6	401	0.002	T=4.24	Post	0.03 (0.15)	-0.24 (0.10)
					Pre	-0.12 (0.15)	-0.01 (0.16)
Cognitive control network							
Left DLPFC, left superior frontal gyrus, medial (BA 9)	-15, 48, 30	351	0.006	T=4.17	Post	0.02 (0.24)	-0.20 (0.26)
					Pre	-0.16 (0.22)	-0.06 (0.24)
Left DLPFC, right hippocampus	27, -42, 9	336	0.008	T=5.28	Post	-0.15 (0.11)	0.07 (0.14)
					Pre	0.05 (0.21)	-0.16 (0.17)
Right DLPFC	…						
DMN							
Left PRE, right middle frontal gyrus, lateral (BA 10/46)	39, 42, 15	243	0.042	T=3.05	Post	-0.07 (0.23)	-0.28 (0.18)
					Pre	-0.31 (0.23)	-0.05 (0.30)
Right PRE	…						
mPFC, right middle occipital/ middle temporal (BA 19/18)	30, -81, 9	588	<0.001	T=4.65	Post	-0.13 (0.18)	0.16 (0.11)
					Pre	0.03 (0.21)	-0.11 (0.22)
PCC, left inferior temporal/middle temporal (BA 20/21)	-39, -30, -15	279	0.024	T=4.72	Post	0.10 (0.20)	-0.23 (0.19)
					Pre	-0.07 (0.20)	0.06 (0.20)

Abbreviations: ACC, anterior cingulate cortex; BA, Brodmann area; DLPFC, dorsolateral prefrontal cortex; DMN, default mode network; FEF, frontal eye field; FWE, Family-wise error; IPS, inferior parietal sulcus; MNI, Montreal Neurological Institute; mPFC, medial prefrontal cortex; PCC, posterior cingulate cortex; PRE, precuneus; Rz, z-transformed Pearson’s correlational coefficient; TPJ, temporoparietal junction; VFC, ventral frontal cortex.

^*a*^The normalized voxel was resampled to the size of isotropic 3mm.

^*b*^The cluster-forming threshold was set at voxel-level *P<*.01.

### Changes in RSFC with Treatment Response

**Figure 4.**
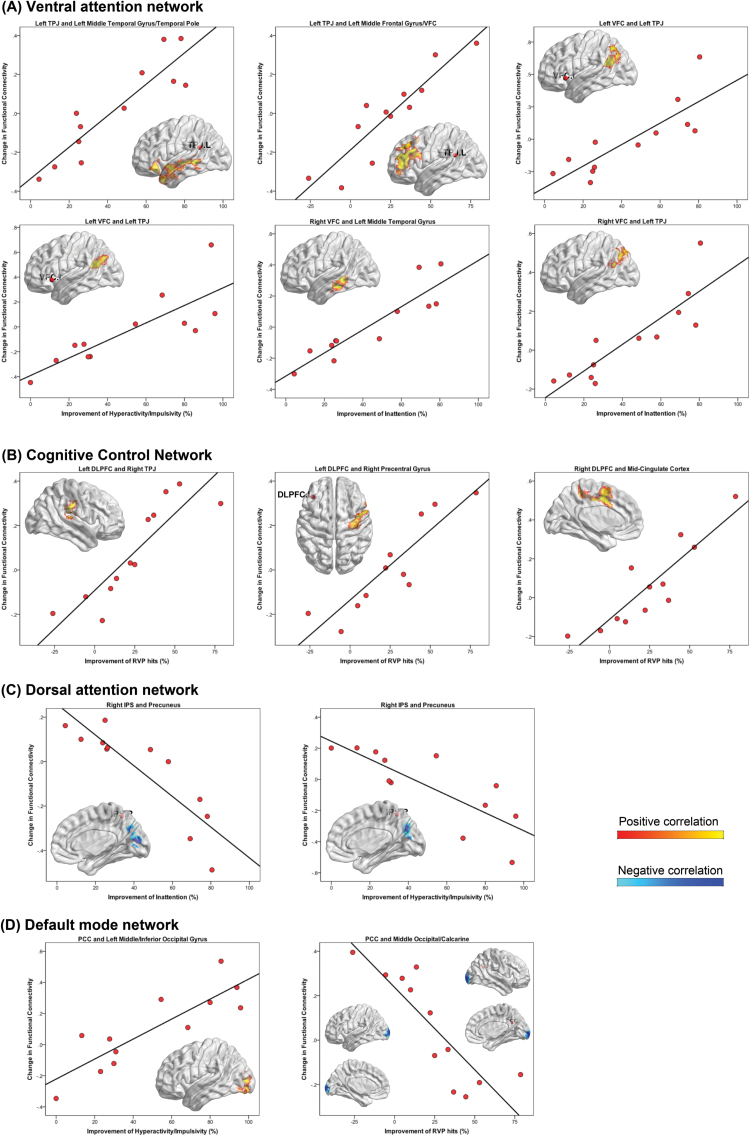
Functional connectivity changes with improvement in clinical symptoms and neuropsychological performances. Regions that showed significant (statistical height threshold *P<*.01, FWE cluster-level corrected *P<*.05) alterations in functional connectivity as symptoms and performances of Rapid Visual Information Processing (RVP) improved, in (A) ventral attention network, (B) cognitive control network, (C) dorsal attention network, and (D) default mode network (DMN). Yellow maps corresponded to positive associations, whereas blue maps represented negative associations. DLPFC,dorsolateral prefrontal cortex; FEF,frontal eye field; PCC,posterior cingulate cortex; PRE,precuneus; TPJ,temporo-parietal junction; VFC,ventral frontal cortex.

As shown in Figure 4 and Table 5, greater reductions in inattention symptoms showed a positive correlation with increased connectivity between the left TPJ and left MTG (*P<*.001), between the left VFC and left TPJ (*P*=.001), between the right VFC and left MTG (*P*=.046), and between the right VFC and left TPJ (*P=*.017) in the ventral attention network. As hyperactivity/impulsivity improved, increased RSFC was observed in the ventral attention network between the left VFC and TPJ (*P*=.014) and in the DMN between the PCC and left middle/inferior occipital gyrus (*P*=.007). We identified negative correlations between symptom improvement and changes in RSFC between the right IPS and PRE in the dorsal attention network (inattention *P=*.022; hyperactivity/impulsivity *P*=.026). Regarding neuropsychological performances, we observed positive correlations of RVP total hits with increased RSFC between the left TPJ and left middle frontal gyrus/VFC in the ventral attention network (*P<*.001); between the left DLPFC and right TPJ (*P=*.019), as well as right precentral gyrus (*P*=.002), respectively; and between the right DLPFC and left mid-cingulate cortex (*P=*.001) in the cognitive control network. With improving RVP performances, we identified less connectivity between the PCC and middle occipital/calcarine in the DMN (*P=*.003). We observed no significant associations between changes in behaviors and RSFC in the affective network in the atomoxetine-treated adults.

**Table 5.** Significant Differences in Functional Connectivity From Multiple Regression with Changes in Clinical Symptoms and Neuropsychological Performances

**Network and Regions**	**MNI Coordinate**	**Cluster Size (voxels**)^*a*^	**Cluster-Level FWE-Corrected *P*** ^*b*^	**T-value**	**Behavioral measures**	**Direction of Correlation**
Ventral attention network						
Left TPJ, left middle temporal/ temporal pole (BA 21/38)	-42, 27, -18	589	<0.001	T=7.98	Inattention	Positive
Left TPJ, left middle frontal gyrus/VFC (BA 46)	-51, 30, 21	647	<0.001	T=9.46	RVP hits	Positive
Left VFC, left posterior TPJ (BA 39)	-48, -48, 24	393	0.001	T=5.53	Inattention	Positive
Left VFC, left posterior TPJ (BA 39)	-45, -48, 21	250	0.014	T=6.29	Hyperactivity/ impulsivity	Positive
Right VFC, left middle temporal gyrus (BA 21)	-57, -30, -15	193	0.046	T=7.91	Inattention	Positive
Right VFC, left posterior TPJ (BA 39)	-36, -54, 21	237	0.017	T=5.73	Inattention	Positive
Dorsal attention network						
Right IPS, precuneus/cuneus (BA 18/7)	0, -84, 12	236	0.022	T=5.06	Inattention	Negative
Right IPS, precuneus/cuneus (BA 7/31)	12, -72, 24	234	0.026	T=4.32	Hyperactivity/ impulsivity	Negative
Cognitive control network						
Left DLPFC, right TPJ (BA 40)	69, -27,15	235	0.019	T=7.40	RVP hits	Positive
Left DLPFC, right precentral gyrus (BA 6)	30, -15, 66	335	0.002	T=6.82	RVP hits	Positive
Right DLPFC, left mid-cingulate	-12, 6, 39	383	0.001	T=10.30	RVP hits	Positive
DMN						
PCC, left middle/inferior occipital (BA 18)	-30, -84, -3	294	0.007	T=5.67	Hyperactivity/ impulsivity	Positive
PCC, middle occipital/calcarine (BA 18)	-9, -102, 3	330	0.003	T=5.94	RVP hits	Negative

^*a*^The normalized voxel was resampled to the size of isotropic 3mm.

^*b*^The cluster-forming threshold was set at voxel-level *P<*.01.

Abbreviations: ACC, anterior cingulate cortex; BA, Brodmann area; DLPFC, dorsolateral prefrontal cortex; DMN, default mode network; FEF, frontal eye field; FWE, Family-wise error; IPS, inferior parietal sulcus; MNI, Montreal Neurological Institute; mPFC, medial prefrontal cortex; PCC, posterior cingulate cortex; PRE, precuneus; RVP, Rapid Visual Information Processing; TPJ, temporoparietal junction; VFC, ventral frontal cortex.

## Discussion

To our best knowledge, this is the first study to incorporate rs-fMRI into a randomized clinical trial of atomoxetine in medication-naïve adults with ADHD. We are the first to display causal relationships between atomoxetine use and a strengthening anticorrelated relationship between the task-positive networks (cognitive control and dorsal attention networks) and DMN in adults with ADHD. Moreover, we provide some evidence to support a modulating effect of atomoxetine on all major neural networks in adults with ADHD.

### Altered RSFC in a Medication-Naïve Cohort of Adult ADHD

Consistent with prior literature (Fair et al., 2010), our results support hypoconnectivity within the DMN in medication-naïve adults with ADHD. Relative to the control group, we also found adults with ADHD had hypoconnectivity between the right FEF and right parahippocampal/fusiform gyrus and between the left FEF and right fusiform/inferior temporal gyrus in the dorsal attention network. The hypoconnected right fusiform/inferior temporal gyrus is located between the anterior and posterior lateral part of the fusiform. Hypoactivity of this region in ADHD is associated with poor inhibition of emotional memory (Depue et al., 2010). The parahippocampal/fusiform gyrus is considered to be implicated in emotion regulation (Frank et al., 2014). Atypical morphometry (Proal et al., 2011) and regional functional homogeneity (Cao et al., 2006) in the region have been reported in ADHD. Our finding of decreased connections of the right fusiform with the dorsal attention network in adults with ADHD is in line with the findings from McCarthy and colleagues (2013). Taken together, our findings suggest that atypical interactions between emotional processes and top-down attention regulation may partially underpin the pathophysiology of ADHD (Castellanos et al., 2006).

Our data demonstrate differential RSFC patterns between ADHD and controls with regards to anticorrelated relationships. We found reduced anticorrelations between the hubs of the cognitive control network and DMN (between the left DLPFC and PRE/PCC, and between the right DLPFC and mPFC, a trend-level significance with FWE-corrected *P*=.053) in adults with ADHD (Castellanos et al., 2008; Hoekzema et al., 2014; Mattfeld et al., 2014). Prior reports suggest that an anticorrelated relationship exists between the DMN and task-positive networks (Fox et al., 2005), and these intrinsic anticorrelated networks subserving opposing functions impact behaviors in normal population (Kelly et al., 2008). Individuals with ADHD are also characterized by reduced anticorrelations among these networks (Castellanos et al., 2008; Tomasi and Volkow, 2012; Hoekzema et al., 2014; Mattfeld et al., 2014). Consistent with this concept, our findings suggest a critical role of atypical default-mode interference underpinning executive dysfunction in ADHD (Sonuga-Barke and Castellanos, 2007), independent of a history of medication exposure. Notably, we found conversed directions in connections between the right FEF (the hub of dorsal attention network) and bilateral DLPFC (the component within the cognitive control network), and between the right FEF and left MTG/angular gyrus (spatially overlapping with the hub of the DMN), in adults with ADHD (prone to negative correlations) and the controls (prone to positive connections), respectively. These findings might hint at an atypically dynamic balance between these 3 major networks in ADHD. Future work implicating fine-grained network analyses, for example, graph-theory approach or functional network connectivity, is needed to delineate the coordination of spontaneous activity both within and between these large-scale networks in ADHD. Taken collectively, our findings suggest an atypical cross talk between major functional brain networks would contribute to brain mechanisms central to ADHD.

Despite the above-mentioned comparable findings, to our surprise, this study did not provide evidence to support previous reports of atypical intrinsic functional brain organization in the affective and ventral attention networks for medication-naïve adults with ADHD (McCarthy et al., 2013). Given that we adopted imaging analyses according to the prior published methods, different exposure to methylphenidate, which has been suggested to modulate resting-state brain function (Li et al., 2013), across studies may partially account for these differences (Qi et al., 2014). The current cohort of high-functioning and medication-naïve adults allowed investigation of RSFC in ADHD independent of the effects from developmental delays, general intellectual dysfunction, or a history of medication use.

### Atomoxetine Treatment Modulates RSFC in Adults with ADHD

Using task-fMRI, the acute pharmacological action of atomoxetine is associated with modulation of the prefrontal regions for adults with ADHD (Cubillo et al., 2014a, 2014b; Nandam et al., 2014). Beyond the prefrontal areas, 2-month treatment with atomoxetine modulates inhibitory control-related activation more extensively in the cortical regions in individuals with ADHD (Schulz et al., 2012; Bush et al., 2013). Our findings extend those of prior task-based reports and indicate that the therapeutic effects of atomoxetine on RSFC in medication-naïve adults with ADHD involve all major brain networks investigated herein. As hypothesized, our results demonstrated that symptomatic and behavioral improvement was associated with enhanced functional connections within the ventral attention and cognitive control networks. Notably, atomoxetine treatment was displayed to strengthen anticorrelations between the DMN and the cognitive control network (ACC/mPFC-left DLPFC pair, left PRE-right DLPFC pair, PCC-left inferolateral temporal cortex) and between the DMN and dorsal attention network (bilateral FEF-orbitofrontal cortex/mPFC pairs). The strengthened anticorrelations between the DMN and dorsal attention network (the PRE-right inferior parietal sulcus pair) were associated with improving clinical symptoms in the atomoxetine-treated adults with ADHD. As anticorrelations between the PCC and middle occipital/calcarine, involved in determining visuospatial orientation (Meadows, 2011) and intrinsically negatively connected with the PCC based on the map calculated by neurosynth.org (Yarkoni et al., 2011), strengthened, better sustained attention was noted in adults treated with atomoxetine. The unique mechanism of atomoxetine should be responsible for these therapeutic effects. According to evidence from the animal study, atomoxetine is commonly thought to exert its therapeutic mechanisms via selective NET binding in the prefrontal regions (Bymaster et al., 2002). Nonetheless, recent in-vivo PET imaging studies in primates suggest that atomoxetine extensively occupies NET in the cortical and subcortical regions (Seneca et al., 2006) and greatly occupies both NET and serotonin transporter at clinically relevant doses (Ding et al., 2014). Moreover, Hahn and colleagues (2012) suggest that neural activity in the DMN is modulated by the serotonin system. Therefore, we boldly postulate that atomoxetine might exert effects on this anticorrelated relationship by inhibiting the norepinephrine system to modulate the task-positive network RSFC while concomitantly affecting the DMN activity via modulating the serotonin system. Future studies that combine in vivo PET and fMRI could directly test whether the therapeutic effects of atomoxetine in the treatment of ADHD depend on NET binding or both NET and serotonin transporter occupancy.

Although we did not find significant differences in the RSFC of the affective and ventral attention networks between adults with ADHD and controls at baseline, atomoxetine treatment still displayed modulating effects on these 2 neural networks. Adults with ADHD exhibited enhanced positive connectivity between the left subgenual ACC and right inferior temporal/middle occipital gyrus in the affective network after treatment with atomoxetine for 8 weeks. The right inferior temporal/middle occipital gyrus is implicated in human emotional face processing (Fusar-Poli et al., 2009), and neuroimaging studies have found hyperactivation (Cortese et al., 2012) and altered white matter structures (Silk et al., 2009) in this region for ADHD. Its enhanced functional couplings with the subgenual ACC after treatment with atomoxetine is probably relevant to a therapeutic effect of atomoxetine on emotional dysregulation in ADHD (Retz et al., 2012). However, we did not observe changes in RSFC in the affective network with treatment response. This may arise from insensitive detection of the changes in emotional regulation in the current behavioral and neuropsychological measures.

In the ventral attention system, we observed increased connections between the anatomical hubs within the network as ADHD symptoms improved. Our finding endorses that this network is implicated in the pathophysiology of ADHD (Cortese et al., 2012; McCarthy et al., 2013; Sripada et al., 2014). The connections between the left middle occipital gyrus and the right TPJ also increased after an 8-week treatment with atomoxetine in adults with ADHD. Moreover, as hyperactivity/impulsivity decreased, the similar region of middle/inferior occipital gyrus showed increased connections with the PCC. This occipital region corresponds to the visual area MT, which functionally interacts with the dorsal attention network to reorient attention (Shulman et al., 2009). Likewise, a recent rs-fMRI study demonstrates hypo-functioning of this region in terms of graph-theoretic network measures in children with ADHD (Xia et al., 2014). Our findings of increased functional interactions of the left middle occipital region with the ventral attention, but not dorsal attention network, and changes in RSFC of the region with the PCC with improving behaviors following atomoxetine treatment indicate more complex pictures of visual function, its regulation by attention system, and their interaction with the DMN in ADHD. Such an assumption warrants further validation from independent samples.

### Methodological Considerations and Limitations

Several caveats regarding methodology should be borne in mind to interpret the findings. First, although a repeated-measures ANOVA test demonstrated improvement in clinical symptoms and cognitive performances in the atomoxetine-treated group, in a stringent sense, there was no statistical significance in therapeutic effects of atomoxetine on behavioral ratings, given no significant treatment×time interaction. Discrepancy in the imaging and behavioral findings may arise from sensitive MRI measures, given that the 8-week treatment duration may be insufficient to reach clinically valid symptom reductions with atomoxetine (Newcorn et al., 2009). On the other hand, despite the fact that the MRI findings were controlled for false positives by excluding baseline differences between treatment conditions and by using stringent cluster-level FWE correction (while using relatively liberal cluster-forming threshold may reduce spatial specificity [Woo et al., 2014]), the small sample size (Friston, 2012) may inflate statistical effects. These may restrain the interpretation of clinical trials and brain-behavior associations and introduce type I/II errors. A larger cohort in the future work could help validate our findings.

Several methodological limitations of rs-fMRI should be acknowledged (see the supplementary Material for detailed discussion about the issues). First, the postplacebo group had higher mean FD than the postatomoxetine group. It may arise from atomoxetine effects on ADHD symptoms. Despite that several strategies were applied to minimize in-scanner head motion impacts, including motion-censure exclusion criteria (excluding participants with the translation, alongside maximum FD, and rotation estimates >1.5mm or 1.5°, respectively), component-based denoising method (Behzadi et al., 2007), alongside motion-composite regression at both individual and group levels (Yan et al., 2013), even a relatively small amount of head motion may still confound the present findings. Another key limitation was the lack of objective measures monitoring the state of wakefulness, rendering the possibility of sleep during the scans. Nonetheless, functional neuroanatomy underpinning unstable wakefulness during typical rs-fMRI experiments does not spatially overlap with the current results (Tagliazucchi and Laufs, 2014). Also, there may be systemic bias between eyes-open and eyes-closed resting-state studies (Castellanos et al., 2013) despite that reliability and consistency of functional connectivity strengths in major neural networks are grossly similar across resting conditions (Patriat et al., 2013). Another caveat is the 6-minute scan lengths during rs-fMRI, which may be sufficient to result in stable estimates of RSFC (Fox et al., 2005; Van Dijk et al., 2010). Nonetheless, we acknowledge that reliability of RSFC can be greatly improved as scan lengths increase (Birn et al., 2013).

Earlier studies employing different preprocessing techniques (Fox et al., 2009; Chai et al., 2012; Kundu et al., 2013) suggest that the anticorrelations (negative connections) observed in rs-fMRI are valid. The CompCor method implemented in the present study also allows interpretation of anticorrelations. However, biological origins still elude the phenomenon. Implications for our findings of baseline disconnectivity and the effects of atomoxetine on interrelationships between the major brain networks in ADHD await further investigation.

Owing to the finite spatial coverage, we excluded the cerebellar regions from the rs-fMRI analyses. However, the cerebellum is structurally and functionally connected with prefrontal and striatal circuits implicated in ADHD (Bostan et al., 2013). Individuals with ADHD have atypical functional connectivity between cerebral-cerebellar networks (Tomasi and Volkow, 2012; Kucyi et al., 2015). Atomoxetine at therapeutic doses has shown to occupy NETs in nonhuman primates (Gallezot et al., 2011) and increase regional cerebral blood flow in the cerebellum in typical adults (Marquand et al., 2012). Future studies should investigate the effects of atomoxetine on the cerebral-cerebellar connectivity in ADHD to further complement our findings.

Another limitation of this study is selection bias of our sample. To increase the internal validity of the sample and explore disease-specific alterations, only participants who were medication naïve and comorbidity free were recruited in the present study. This makes generalization of the current findings to the “real-world” clinical settings indistinct, given the high comorbidity and medication exposure rates in an adult cohort of ADHD (Biederman et al., 2006). Lastly, while the focus on the major 5 predefined neural networks based on the published evidence (McCarthy et al., 2013; Mattfeld et al., 2014) could facilitate direct comparisons across studies, this study leaves atomoxetine effects on other brain networks unexamined. Future work employing both seed-based and data-driven approaches (eg, independent component analysis or multivariate analysis) could complement the present study.

## Conclusions

This study shows the atypical natures of relationships between functional brain networks and of connectivity within dorsal attention network and DMN in medication-naïve adults with ADHD. We further provide evidence for a mechanism by which atomoxetine therapy strengthens an anticorrelated relationship between the task-positive networks and DMN and modulates RSFC across all major brain networks in medication-naïve adults with ADHD. Our results support the idea that atypical DMN task-positive networks cross talk may contribute to the pathophysiology of ADHD. Strengthening this relationship following atomoxetine treatment suggests an important pathway through which atomoxetine may improve ADHD.

## Statement of Interest

Dr. Lin has received grant or research support from National Taiwan University Hospital. Dr. Gau has received grant or research support from the National Science Council, Taiwan, the National Health Research Institute, Taiwan, and National Taiwan University Hospital. All authors declare no biomedical financial interests or potential conflicts of interest.

## Supplementary Material

supplementary Material

## Notes

**ClinicalTrials.gov number:** NCT00917371; http://clinicaltrials.gov/show/NCT00917371.
