# Supplementary material for: Atomoxetine Treatment Strengthens an Anti-Correlated Relationship between Functional Brain Networks in Medication-Naïve Adults with Attention-Deficit Hyperactivity Disorder: A Randomized Double-Blind Placebo-Controlled Clinical Trial
Source: Int J Neuropsychopharmacol. 2015 Sep 16;19(3):pyv094. doi: 10.1093/ijnp/pyv094 (PMC4815465; doi:10.1093/ijnp/pyv094)

**Supplementary Introduction**

**Selection of the Five Predefined Networks**

The first network is the default mode network (DMN), which primarily consists of the precuneus/posterior cingulate cortex (PCC), medial prefrontal cortex (mPFC), angular gyrus, lateral temporal cortex, and hippocampus formation. The DMN shows strong coherence in activity at rest and establishes the baseline to reflect intrinsic brain function, and its activity is attenuated during effortful cognitive task (Buckner et al., 2008). It has been associated with non-goal–directed state and may mediate “mind-wandering” mental activity (Mason et al., 2007). Previous resting-state studies demonstrated reduced connectivity between PCC, mPFC, and other loci within DMN (Uddin et al., 2008; Fair et al., 2010) and reduced antiphase relationship between the task-positive networks (Castellanos et al., 2008; Sun et al., 2012; Hoekzema et al., 2013; Mattfeld et al., 2014) in individuals with attention-deficit hyperactivity disorder (ADHD). These 2 specific interactions in the DMN have been exhibited as dissociated in adults who were persistent in versus remitted from childhood diagnoses of ADHD (Mattfeld et al., 2014). However, McCarthy and colleagues disparately found hyperconnectivity within the DMN. We set out to confirm alterations in DMN RSFC as demonstrated in the previous studies in medication naïve adults with ADHD.

The second network involves the cognitive control network (Vincent et al., 2008), which is composed of the anterior prefrontal cortex, dorsolateral prefrontal cortex (DLPFC), inferior frontal gyrus, dorsal anterior cingulate cortex (ACC), anterior insula/frontal operculum, anterior inferior parietal lobule, inferolateral temporal cortex, and caudate (Vincent et al., 2008; Yeo et al., 2011; Geranmayeh et al., 2014). It has been suggested that the cognitive control network underpins executive control during goal attainment and facilitates flexible and optimal decision making by integrating information from internal cognition and external attention (Menon, 2011). Previous structural imaging studies (Valera et al., 2007; Nakao et al., 2011) have consistently reported abnormal morphometry in the DLPFC and caudate and in the prefrontal, cingulate, and parietal areas in ADHD. Recent meta-analyses (Cortese et al., 2012; Hart et al., 2013) on task-fMRI studies have documented hypoactivation of the components within the cognitive control network in ADHD across executive tasks. Rs-fMRI studies using various analytic methods also suggest involvement of the cognitive control network in ADHD (Cao et al., 2006; Zang et al., 2007; Wang et al., 2009; Qiu et al., 2011; Posner et al., 2013).

The third and fourth networks implicate the dorsal and ventral attention networks, which form the attention regulatory system of the brain (Corbetta et al., 2008). The dorsal attention network mainly involves the frontal eye field and intraparietal sulcus to enable top-down control of attention and has been implicated in the etiology of ADHD (Castellanos and Proal, 2012; Cortese et al., 2012; McCarthy et al., 2013; Elton et al., 2014). The ventral attention network uses the temporoparietal junction, ventral frontal cortex, and supramarginal gyrus to monitor salient stimuli and reorient attention (Corbetta et al., 2008). Recent studies demonstrated hypoactivation during reorienting tasks (Cortese et al., 2012) and resting-state hypoconnectivity (McCarthy et al., 2013) within the ventral attention network in ADHD. We set out to confirm that aberrant RSFC in the dorsal and ventral attention networks would contribute to brain mechanisms central to ADHD.

The fifth network is the affective network. This network includes the subgenual ACC (the affective subdivision of ACC [Beckmann et al., 2009]), amygdala, nucleus accumbens, hypothalamus, anterior insula, hippocampus, and orbitofrontal cortex and is involved in emotional processing and regulation (Sheline et al., 2010). Emotional dysregulation is highly prevalent in ADHD throughout the individual’s lifespan (Shaw et al., 2014), and adults with ADHD are commonly comorbid with the affective disorder (Biederman et al., 2006). Previous functional neuroimaging studies demonstrate that ADHD patients have abnormal activities within the affective network regions (Shaw et al., 2014). Likewise, increased resting-state functional connectivity (RSFC) in the affective network has been found in depression (Sheline et al., 2010) and ADHD (McCarthy et al., 2013; Hulvershorn et al., 2014) patients. By using the same subgenual ACC seed as in the previous studies (Sheline et al., 2010; McCarthy et al., 2013), we anticipated atypical RSFC in adults with ADHD, relative to healthy controls, within this network.

**SUPPLEMENTARY METHODS**

**Measures**

**Adult ADHD Self-Report Scale—**The Adult ADHD Self-Report Scale (ASRS), an 18-question scale, was developed in conjunction with the revision of the World Health Organization Composite International Diagnostic Interview. The ASRS consists of 2 subscales, inat­tention and hyperactivity-impulsivity (9 items, respectively), according to the 18 *DSM-IV* ADHD symptom crite­ria. Each item asks how often a symptom occurred during the last 6 months on a 5-point Likert scale: 0 = never, 1 = rarely, 2 = sometimes, 3 = often, and 4 =  ery often. The psychometric properties of the Chinese ASRS have been established in a sample of 4329 Taiwanese young adults (Yeh et al., 2008). It has been used extensively in studies on adults with ADHD in Taiwan (Ni et al., 2013a, 2013b).

**Rapid Visual Information Processing—**The Rapid Visual Information Processing (RVP) task, a 4-minute visual continuous performance task, is designed to assess visual sustained attention capacity using numbers as stimuli. Digits (ranging from 2 to 9) appeared one at a time (100 digits/min) in the center of a screen in random order. Participants were asked to press a response pad when they detected any 1 of 3 number sequences (3–5–7, 2–4–6, 4–6–8). “Total hits” (the number of total corrected responses) was presented as the main outcome to index attention capacity (Gau and Huang, 2014).

**MRI Parameters—**The imaging parameters were 180 echo planar imaging (EPI) volumes; TR = 2000 ms; TE = 24 ms; flip angle = 90˚; field of view (FOV) = 256 × 256 mm^2^; matrix size = 64 × 64; 34 axial slices acquired in an interleaved descending order; slice thickness = 3 mm; voxel size = 4 × 4 × 3 mm^3^; imaging plane being parallel to the anterior commissure–posterior commissure (AC–PC) image plane. For spatial normalization, a high-resolution T1-weighted anatomical image was also acquired (MPRAGE, TR = 2000 ms; TE = 2.98 ms; TI = 900 ms; flip angle = 9˚; FOV = 256 × 256 mm^2^; matrix size = 256 × 256; isotropic voxel size = 1 mm). The scan order began with rs-fMRI, followed by T1 anatomical image.

**Functional Imaging Preprocessing—**Data preprocessing, including slice timing, realignment, coregistration, segmentation, normalization, and smoothing, was performed using DPARSF toolbox (Yan and Zhang, 2010) based on Statistical Parametric Mapping (SPM8). The first 5 EPI volumes were discarded to allow for signal equilibration. Functional images were slice-timed, and each volume was realigned to the first image volume using a least-squares minimization and a 6-parameter (rigid-body) spatial transformation. Translation and rotation estimates were required to be <1.5 mm or 1.5°, respectively. Motion-corrected EPI images were then registered to structural scans, normalized to a Montreal Neurological Institute (MNI) template with isotropic 3-mm voxel via the gray matter segment, and smoothed with a 8-mm FWHM Gaussian kernel.

Prior to temporal filtering, we calculated the frame-wise displacement (FD) of in-scanner head motion based on the measures derived from Jenkinson and colleagues (2002; Yan et al., 2013) to ensure all EPI data did not exhibit maximum FD > 1.5 mm. We further used an artifact detection toolbox, ART (<http://www.nitrc.org/projects/artifact_detect>), to identify outlier data points defined as volumes that exceeded a composite movement threshold of 0.5 mm FD. The identified outlier time points would be regressed out at the first level (see the following paragraph). The composite movement measure implemented in ART was calculated by converting the 3 rotation and 3 translation parameters into parameters reflecting the trajectories of 6 points located on the center of each of the faces of a bounding box around the brain.

Spatial coverage of normalized EPI data was inspected for each participant to ensure all cortical regions were covered. The smoothed fMRI data of limited in-scanner motion and adequate spatial coverage were further denoised using CONN toolbox v.13p (Whitfield-Gabrieli and Nieto-Castanon, 2012). Spurious sources of nonneuronal noise were accounted for through a component-based (anatomical CompCor) approach (Behzadi et al., 2007). The first 5 principal components of the signals from the WM and CSF ROIs (anatomical masks derived from the prior segmentation step), 6 realign parameters, alongside their first-order temporal derivatives, and outliers (detected by ART), linear detrending were included as regressors in the first-level denoising regression model. All of the regressors were filtered before performing the denoising regression (Hallquist et al., 2013). Despiking (squashing of extreme time series outliers) was performed before regression and temporally band-pass filtering (0.01-0.08 Hz) was performed simultaneously with regression (‘Simult’).

**Specification of Settings in Statistical Models in SPM8**

For baseline comparisons, we analyzed rs-fMRI data in SPM8 using 2-sample *t* tests (‘independence’ and ‘unequal variance’ specified in second-level analysis settings) to determine significant differences in RSFC between ADHD and controls. For treatment effects, we entered each ADHD participant's seed-based connectivity map into a 2 × 2 repeated-measure factorial model using SPM8. We treated time as a repeated measure with 2 levels: pre- and posttreatment scans (‘no independence’ and ‘equal variance’ for ‘time’ factor in second-level analysis settings); we used treatment as a between-group factor with 2 levels: atomoxetine and placebo (‘independence’ and ‘unequal variance’ for ‘treatment’ factor in second-level analysis settings).

**SUPPLEMENTARY DISCUSSION**

**Methodological Considerations Regarding Resting-State fMRI**

Although there was no significant difference observed in mean FD, maximum FD, and numbers of outliers between the groups at baseline, the postplacebo group had higher mean FD than the postatomoxetine group. It may be owing to the effects of atomoxetine on ADHD core symptoms. Therefore, several strategies were applied to minimize in-scanner head motion impacts on RSFC in the present study. We restricted the translation, alongside maximum FD, and rotation estimates to be <1.5mm or 1.5°, respectively. We opted for CompCor as the denoising method, which has effective capacity to correct head motion and physiological noises (Behzadi et al., 2007). Moreover, spiking outliers were regressed out at the first-level analyses, and individual mean FD was included as a covariate in the group-level analyses (Yan et al., 2013). However, we acknowledge that even a relatively small amount of in-scanner head motion may still confound the present findings of RSFC. Future work incorporating state-of-art image acquisition method (Kundu et al., 2013) or use of navigator scans to directly measure motion (Reuter et al., 2015) could solve this nagging issue.

A key limitation of the current study was the lack of objective measures monitoring the state of wakefulness, rendering possibilities of sleep during scan. Asleep/awake state affects RSFC in a regionally specific pattern, mostly at subcortical areas (thalamus and hypothalamus), alongside sensory and motor regions in typical adults (Tagliazucchi and Laufs, 2014) and children with autism (Di Martino et al., 2015), while intrinsic functional connectivity architectures are grossly similar in higher-order regions in autism (Di Martino et al., 2015), toddlers (Redcay et al., 2007), and adults (Fukunaga et al., 2006; Tagliazucchi and Laufs, 2014) across states. Another caveat would extend to systemic bias between eyes-open and eyes-closed resting-state studies, as reviewed in Castellanos et al. (2013). Nonetheless, a recent study (Tagliazucchi and Laufs, 2014) has suggested participants with eyes-open scan do not fall asleep significantly less than those with eyes closed. Furthermore, reliability and consistency of functional connectivity strengths in major neural networks are grossly similar across resting conditions (Patriat et al., 2013). Collectively, although functional neuroanatomy underpinning unstable wakefulness during typical rs-fMRI experiments does not spatially overlap with the current results, future studies should investigate the impacts of eye-open/closed conditions and awake/asleep states in relation to atomoxetine effects on RSFC in ADHD.

We acknowledge that reliability and similarity of resting-state functional connectivity can be greatly improved as the scan lengths increase (Birn et al., 2013). However, prior studies have shown that around 6 minutes duration of acquisition results in stable estimates of intrinsic connectivity networks (Fox et al., 2005; Van Dijk et al., 2010); thereby, most of current resting-state fMRI studies acquire such duration of data to obtain estimates of connectivity. Using the scrubbing strategy to address motion-related artifacts, a 5-minute (Power et al., 2012) or 4-minute (Satterthwaite et al., 2013) minimum criteria is applied to ensure reliable functional connectivity estimates. Similarly, good inter-session reliability is found for functional homogeneity analyses with scan durations as brief as 5 minutes (Zuo et al., 2013). Using the root-mean-square deviation measuring the mean difference between connectivity values from different fMRI scans of the same individual, Birn and colleagues (2013) have found improvements in root-mean-square deviation plateau around 7 to 8 minutes, which is consistent with work by Van Dijk et al. (2010). In summary, we acknowledge that longer scan lengths could result in higher inter- or intra-session reliability of intrinsic functional connectivity networks. Nonetheless, balancing the trade-off of scan lengths, motion confounds, and practicability, we argue that obtaining the current scan lengths (6 minutes) of rs-fMRI could result in satisfyingly reliable functional connectivity estimates.

**REFERENCES**

Beckmann M, Johansen-Berg H, Rushworth MF (2009) Connectivity-based parcellation of human cingulate cortex and its relation to functional specialization. J Neurosci 29:1175–1190.

Behzadi Y, Restom K, Liau J, Liu TT (2007) A component based noise correction method (CompCor) for BOLD and perfusion based fMRI. NeuroImage 37:90–101.

Biederman J, Monuteaux MC, Mick E, Spencer T, Wilens TE, Silva JM, Snyder LE, Faraone SV (2006) Young adult outcome of attention deficit hyperactivity disorder: a controlled 10-year follow-up study. Psychol Med 36:167–179.

Buckner RL, Andrews-Hanna JR, Schacter DL (2008) The brain's default network: anatomy, function, and relevance to disease. Ann N Y Acad Sci 1124:1–38.

Cao Q, Zang Y, Sun L, Sui M, Long X, Zou Q, Wang Y (2006) Abnormal neural activity in children with attention deficit hyperactivity disorder: a resting-state functional magnetic resonance imaging study. Neuroreport 17:1033–1036.

Castellanos FX, Proal E (2012) Large-scale brain systems in ADHD: beyond the prefrontal-striatal model. Trends Cogn Sci 16:17–26.

Castellanos FX, Margulies DS, Kelly C, Uddin LQ, Ghaffari M, Kirsch A, Shaw D, Shehzad Z, Di Martino A, Biswal B, Sonuga-Barke EJ, Rotrosen J, Adler LA, Milham MP (2008) Cingulate-precuneus interactions: a new locus of dysfunction in adult attention-deficit/hyperactivity disorder. Biol Psychiatry 63:332–337.

Corbetta M, Patel G, Shulman GL (2008) The reorienting system of the human brain: from environment to theory of mind. Neuron 58:306–324.

Cortese S, Kelly C, Chabernaud C, Proal E, Di Martino A, Milham MP, Castellanos FX (2012) Toward systems neuroscience of ADHD: a meta-analysis of 55 fMRI studies. Am J Psychiatry 169:1038–1055.

Elton A, Alcauter S, Gao W (2014) Network connectivity abnormality profile supports a categorical-dimensional hybrid model of ADHD. Hum Brain Mapp 35:4531–4543.

Fair DA, Posner J, Nagel BJ, Bathula D, Dias TG, Mills KL, Blythe MS, Giwa A, Schmitt CF, Nigg JT (2010) Atypical default network connectivity in youth with attention-deficit/hyperactivity disorder. Biol Psychiatry 68:1084–1091.

Gau SS, Huang WL (2014) Rapid visual information processing as a cognitive endophenotype of attention deficit hyperactivity disorder. Psychol Med 44:435–446.

Geranmayeh F, Wise RJ, Mehta A, Leech R (2014) Overlapping networks engaged during spoken language production and its cognitive control. J Neurosci 34:8728–8740.

Hallquist MN, Hwang K, Luna B (2013) The nuisance of nuisance regression: spectral misspecification in a common approach to resting-state fMRI preprocessing reintroduces noise and obscures functional connectivity. NeuroImage 82:208–225.

Hart H, Radua J, Nakao T, Mataix-Cols D, Rubia K (2013) Meta-analysis of functional magnetic resonance imaging studies of inhibition and attention in attention-deficit/hyperactivity disorder: exploring task-specific, stimulant medication, and age effects. JAMA Psychiatry 70:185–198.

Hoekzema E, Carmona S, Ramos-Quiroga JA, Richarte Fernandez V, Bosch R, Soliva JC, Rovira M, Bulbena A, Tobena A, Casas M, Vilarroya O (2013) An independent components and functional connectivity analysis of resting state fMRI data points to neural network dysregulation in adult ADHD. Hum Brain Mapp.

Hulvershorn LA, Mennes M, Castellanos FX, Di Martino A, Milham MP, Hummer TA, Roy AK (2014) Abnormal amygdala functional connectivity associated with emotional lability in children with attention-deficit/hyperactivity disorder. J Am Acad Child Adolesc Psychiatry 53:351–361 e351.

Jenkinson M, Bannister P, Brady M, Smith S (2002) Improved optimization for the robust and accurate linear registration and motion correction of brain images. NeuroImage 17:825–841.

Lancaster JL, Tordesillas-Gutierrez D, Martinez M, Salinas F, Evans A, Zilles K, Mazziotta JC, Fox PT (2007) Bias between MNI and Talairach coordinates analyzed using the ICBM-152 brain template. Hum Brain Mapp 28:1194–1205.

Mason MF, Norton MI, Van Horn JD, Wegner DM, Grafton ST, Macrae CN (2007) Wandering minds: the default network and stimulus-independent thought. Science 315:393–395.

Mattfeld AT, Gabrieli JD, Biederman J, Spencer T, Brown A, Kotte A, Kagan E, Whitfield-Gabrieli S (2014) Brain differences between persistent and remitted attention deficit hyperactivity disorder. Brain.

McCarthy H, Skokauskas N, Mulligan A, Donohoe G, Mullins D, Kelly J, Johnson K, Fagan A, Gill M, Meaney J, Frodl T (2013) Attention network hypoconnectivity with default and affective network hyperconnectivity in adults diagnosed with attention-deficit/hyperactivity disorder in childhood. JAMA Psychiatry 70:1329–1337.

Menon V (2011) Large-scale brain networks and psychopathology: a unifying triple network model. Trends Cogn Sci 15:483–506.

Nakao T, Radua J, Rubia K, Mataix-Cols D (2011) Gray matter volume abnormalities in ADHD: voxel-based meta-analysis exploring the effects of age and stimulant medication. Am J Psychiatry 168:1154–1163.

Ni HC, Lin YJ, Gau SS, Huang HC, Yang LK (2013a) An open-label, randomized trial of methylphenidate and atomoxetine treatment in adults With ADHD. J Atten Disord.

Ni HC, Shang CY, Gau SS, Lin YJ, Huang HC, Yang LK (2013b) A head-to-head randomized clinical trial of methylphenidate and atomoxetine treatment for executive function in adults with attention-deficit hyperactivity disorder. Int J Neuropsychopharmacol 16:1959–1973.

Posner J, Rauh V, Gruber A, Gat I, Wang Z, Peterson BS (2013) Dissociable attentional and affective circuits in medication-naive children with attention-deficit/hyperactivity disorder. Psychiatry Res

Qiu MG, Ye Z, Li QY, Liu GJ, Xie B, Wang J (2011) Changes of brain structure and function in ADHD children. Brain Topogr 24:243–252.

Shaw P, Stringaris A, Nigg J, Leibenluft E (2014) Emotion dysregulation in attention deficit hyperactivity disorder. Am J Psychiatry 171:276–293.

Sheline YI, Price JL, Yan Z, Mintun MA (2010) Resting-state functional MRI in depression unmasks increased connectivity between networks via the dorsal nexus. Proc Natl Acad Sci U S A 107:11020–11025.

Sun L, Cao Q, Long X, Sui M, Cao X, Zhu C, Zuo X, An L, Song Y, Zang Y, Wang Y (2012) Abnormal functional connectivity between the anterior cingulate and the default mode network in drug-naive boys with attention deficit hyperactivity disorder. Psychiatry Res 201:120–127.

Uddin LQ, Kelly AM, Biswal BB, Margulies DS, Shehzad Z, Shaw D, Ghaffari M, Rotrosen J, Adler LA, Castellanos FX, Milham MP (2008) Network homogeneity reveals decreased integrity of default-mode network in ADHD. J Neurosci Methods 169:249–254.

Valera EM, Faraone SV, Murray KE, Seidman LJ (2007) Meta-analysis of structural imaging findings in attention-deficit/hyperactivity disorder. Biol Psychiatry 61:1361–1369.

Vincent JL, Kahn I, Snyder AZ, Raichle ME, Buckner RL (2008) Evidence for a frontoparietal control system revealed by intrinsic functional connectivity. J Neurophysiol 100:3328–3342.

Wang L, Zhu C, He Y, Zang Y, Cao Q, Zhang H, Zhong Q, Wang Y (2009) Altered small-world brain functional networks in children with attention-deficit/hyperactivity disorder. Hum Brain Mapp 30:638–649.

Whitfield-Gabrieli S, Nieto-Castanon A (2012) Conn: a functional connectivity toolbox for correlated and anticorrelated brain networks. Brain Connect 2:125–141.

Yan CG, Zhang YF (2010) DPARSF: a MATLAB toolbox for "pipeline" data analysis of resting-state fMRI. Front Syst Neurosci 4:13.

Yan CG, Cheung B, Kelly C, Colcombe S, Craddock RC, Di Martino A, Li Q, Zuo XN, Castellanos FX, Milham MP (2013) A comprehensive assessment of regional variation in the impact of head micromovements on functional connectomics. NeuroImage 76:183–201.

Yeh CB, Gau SS, Kessler RC, Wu YY (2008) Psychometric properties of the Chinese version of the adult ADHD Self-report Scale. Int J Methods Psychiatr Res 17:45–54.

Yeo BT, Krienen FM, Sepulcre J, Sabuncu MR, Lashkari D, Hollinshead M, Roffman JL, Smoller JW, Zollei L, Polimeni JR, Fischl B, Liu H, Buckner RL (2011) The organization of the human cerebral cortex estimated by intrinsic functional connectivity. J Neurophysiol 106:1125–1165.

Zang YF, He Y, Zhu CZ, Cao QJ, Sui MQ, Liang M, Tian LX, Jiang TZ, Wang YF (2007) Altered baseline brain activity in children with ADHD revealed by resting-state functional MRI. Brain Dev 29:83–91.

**Supplementary Table 1. Pearson’s Correlations of Seed-ROI Connectivity with Age and Full-Scale IQ**

| **Control-ADHD** | | **Left FEF, Right Fusiform Gyrus/Inferior Temporal Gyrus (BA 20/37)** | **Right FEF, Right Parahippocampal/Fusiform Gyrus** | **Right FEF, Right Middle Frontal Gyrus (BA 8)** | **Left DLPFC, Precuneus/Posterior Cingulate Gyrus (BA 31/30)** | **Right DLPFC, Medial Prefrontal Cortex (BA 32/10)** | **Left PRE, Right Middle Temporal/Fusiform Gyrus (BA 20/21)** | **Right FEF, Left Middle Temporal/Angular Gyrus (BA 39)** | **Right FEF, Left Middle Frontal Gyrus (BA 8)** |
| --- | --- | --- | --- | --- | --- | --- | --- | --- | --- |
| **Age** | **R** | -0.029 | -0.040 | 0.056 | -0.276 | -0.213 | 0.264 | 0.066 | 0.109 |
|  | **P** | 0.847 | 0.785 | 0.705 | 0.058 | 0.147 | 0.070 | 0.658 | 0.462 |
| **Full**  **IQ** | **r** | 0.063 | 0.076 | 0.111 | 0.021 | 0.084 | -0.166 | -0.091 | -0.019 |
|  | **p** | 0.669 | 0.608 | 0.453 | 0.889 | 0.571 | 0.260 | 0.537 | 0.896 |

| **Treatment × time interaction** | | Left subgenual ACC, right inferior temporal/middle occipital gyrus | Right TPJ, left middle occipital gyrus (BA 18/19) | Left FEF, left orbitofrontal cortex/mPFC (BA 11/10) | Right FEF, orbitofrontal cortex/mPFC (BA 11/10) | Left DLPFC, left superior frontal gyrus, medial (BA 9) | Left DLPFC, right hippocampus | Left PRE, right middle frontal gyrus, lateral (BA 10/46) | mPFC, right middle occipital/middle temporal (BA 19/18) | PCC, left inferior temporal/middle temporal (BA 20/21) |
| --- | --- | --- | --- | --- | --- | --- | --- | --- | --- | --- |
| **Age** | **r** | 0.033 | 0.033 | -0.074 | -0.093 | -0.124 | 0.091 | -0.059 | 0.052 | -0.013 |
|  | **p** | 0.822 | 0.824 | 0.615 | 0.528 | 0.401 | 0.539 | 0.689 | 0.727 | 0.927 |
| **Full**  **IQ** | **r** | 0.087 | 0.192 | -0.109 | 0.132 | 0.084 | 0.069 | -0.048 | -0.123 | -0.113 |
|  | **p** | 0.556 | 0.192 | 0.460 | 0.370 | 0.573 | 0.640 | 0.748 | 0.406 | 0.445 |

**Supplementary Table 2. Symptom and Neuropsychological Performance Changes from Baseline to Weeks 8 to 10 in Double-blind, Placebo-controlled Clinical Trial**

|  | **Atomoxetine (n = 12)** | | | | |  | **Placebo (n = 12)** | | | | **Interactions** | |
| --- | --- | --- | --- | --- | --- | --- | --- | --- | --- | --- | --- | --- |
|  | **Baseline** | **Weeks 8-10** | **Repeated-measures ANOVA** | | |  | **Baseline** | **Weeks 8-10** | **Repeated-measures ANOVA** | | **Treatment × time (mixed ANOVA)** | |
|  | **Mean (SD)** | **Mean (SD)** | **F _(1,11)_** | | ***P* value** |  | **Mean (SD)** | **Mean (SD)** | **F _(1,11)_** | ***P* value** | **F _(1,22)_** | ***P* value** |
| Clinical symptoms  Adult Self Report Scale | | | | | | | | | | | | |
| Inattention | 26.42 (6.53) | 13.75 (5.75) | | 19.53 | .001 |  | 27.67 (5.69) | 19.08 (11.11) | 13.45 | 0.004 | 1.22 | 0.282 |
| Hyperactivity-impulsivity | 19.17 (6.93) | 8.42 (6.16) | | 15.01 | .003 |  | 20.67 (6.72) | 15.75 (11.80) | 3.87 | 0.075 | 2.44 | 0.133 |
| Cambridge Neuropsychological Test Automated Battery  RVP | | | | | | | | | | | | |
| Total hits | 18.75 (2.67) | 22.75 (3.33) | | 8.8 | .013 |  | 19.00 (4.11) | 19.92 (4.60) | 0.45 | 0.515 | 2.59 | 0.122 |
| Vital signs | | | | | | | | | | | | |
| Systolic pressure | 116.42 (13.26) | 117.17 (14.84) | | 0.03 | .875 |  | 120.58 (14.95) | 120.25 (21.85) | 0.00 | 0.953 | 0.02 | 0.882 |
| Diastolic pressure | 76.75 (11.59) | 78.00 (9.59) | | 0.16 | .694 |  | 77.58 (8.66) | 77.67 (12.90) | 0.00 | 0.983 | 0.06 | 0.812 |
| Heart Rate | 74.25 (10.63) | 81.00 (20.06) | | 2.78 | .124 |  | 68.67 (10.39) | 75.92 (12.51) | 8.28 | 0.015 | 0.01 | 0.917 |

**Supplementary Table 3. In-Scanner Head Motion of Study Participants**

|  | **Controls** | **Adult ADHD** | | | | |  | **Comparisons** | | | | | |
| --- | --- | --- | --- | --- | --- | --- | --- | --- | --- | --- | --- | --- | --- |
|  |  | **All** | **Atomoxetine** | | **Placebo** | |  | **ADHD vs Control** | **Atomoxetine vs Placebo** | | **Repeated-measures ANOVA** | | **Treatment × time (mixed ANOVA)** |
|  | (n = 24) | (n = 24) | (n = 12) | | (n = 12) | |  |  |  |  |  |  |  |
| Mean framewise displacement (mm), mean (SD)*^a^* | 0.129 (0.053) | 0.116 (0.055) | Pre | 0.100 (0.039) | Pre | 0.133 (0.066) |  | F = 0.64, *P* = .429 | Pre | F = 2.34, *P* = .140 | ATX | F = 0.98, *P* = .343 | F = 1.25, *P* = .276 |
|  |  |  | Post | 0.107 (0.032) | Post | 0.157 (0.063) |  |  | Post | F = 6.06, *P* = .022 | PLA | F = 3.25, p = 0.099 |  |
| Maximum framewise displacement (mm), mean (SD)*^a^* | 0.397 (0.109) | 0.456 (0.231) | Pre | 0.363 (0.176) | Pre | 0.549 (0.248) |  | F = 1.29, *P* = .262 | Pre | F = 4.52, *P* = .045 | ATX | F = 3.39, *P* = .093 | F = 0.002, *P* = .989 |
|  |  |  | Post | 0.485 (0.289) | Post | 0.669 (0.536) |  |  | Post | F = 1.10, *P* = .307 | PLA | F = 0.57, *P* = .465 |  |
| *N* outliers*^b^* | 8.63 (9.66) | 8.42 (10.38) | Pre | 6.08 (9.33) | Pre | 10.75 (11.25) |  | F = 0.005 *P* = .943 | Pre | F = 1.22, *P* = .281 | ATX | F = 0.01, p = 0.761 | F = 0.04, *P* = .854 |
|  |  |  | Post | 7.00 (5.43) | Post | 10.92 (7.51) |  |  | Post | F = 2.14, *P* = .157 | PLA | F = 0.004, *P* = .953 |  |

Abbreviations: ATX,  atomoxetine; PLA,  placebo; Post,  posttreatment (week 8); Pre,  pretreatment (baseline).

*^a^*Framewise displacement (volume to volume displacement) was derived from Jenkinson et al. (2002).

*^b^*Framewise displacements >0.5 mm, based on the motion composite calculated by ART toolbox.

Supplementary Figure 1. Distributions of measures of in-scanner head motion.

**
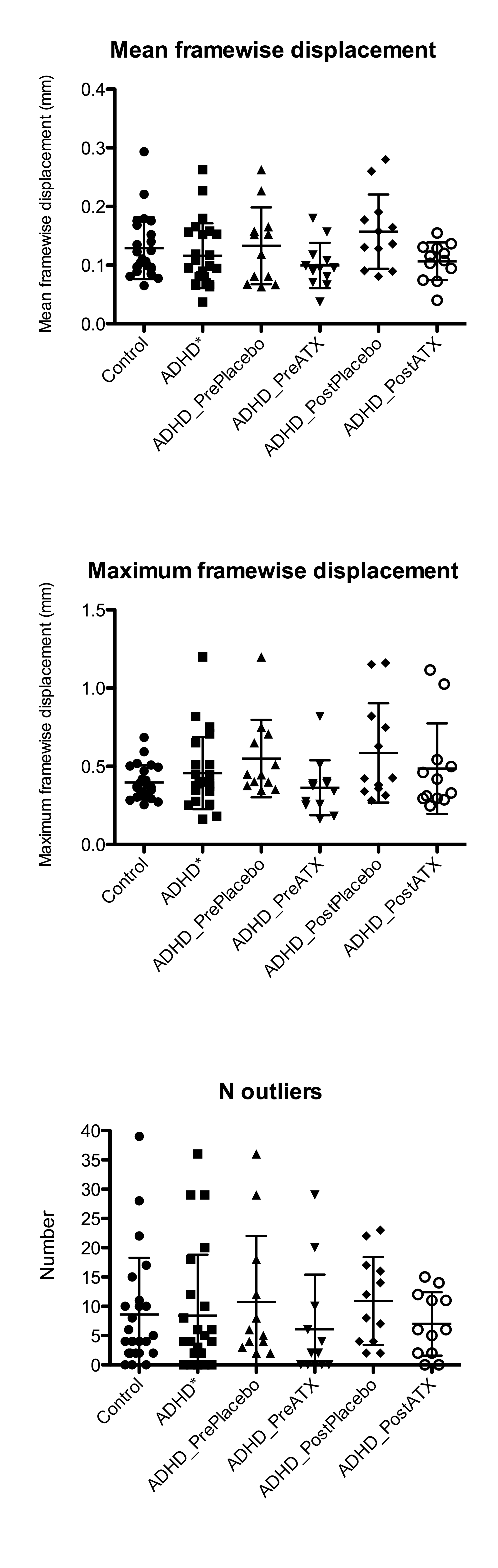
**

Bars represent 1 standard deviation.

*: at baseline

Abbreviations: ADHD, attention deficit hyperactivity disorder; ATX, atomoxetine; Post, posttreatment (weeks 8-10); Pre, pretreatment (baseline).

Supplementary Figure 2. Spatial extents and main hubs of 5 neural networks (identified by using one-sample *t* test in the control group; statistical height threshold *P* < 0.01, FWE cluster-level corrected *P* < 0.05).


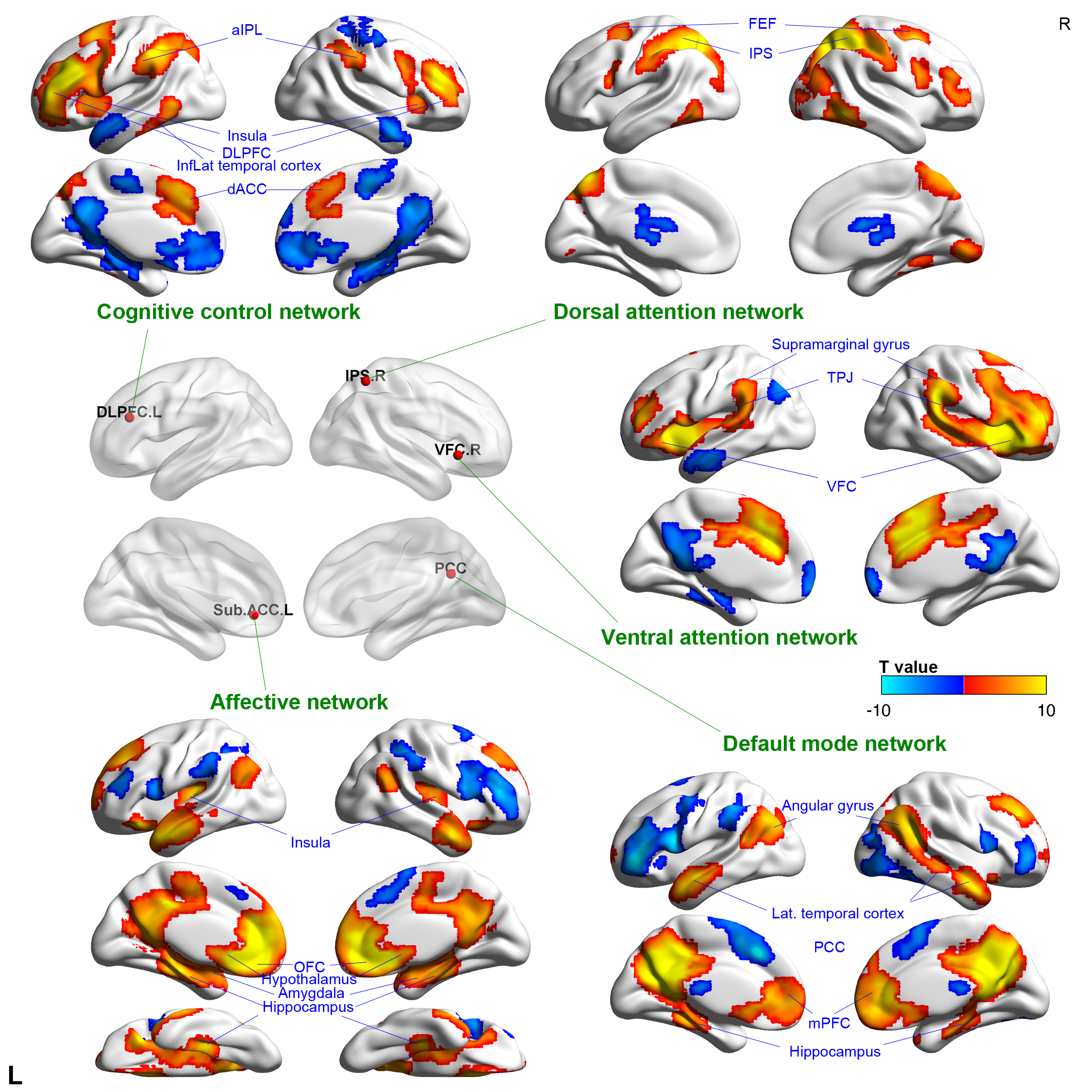


Abbreviations: aIPL,  anterior inferior parietal lobule; dACC,  dorsal anterior cingulate cortex; DLPFC,  dorsolateral prefrontal cortex; FEF,  frontal eye field; InfLat, inferior lateral; IPS,  inferior parietal sulcus; L,  left; Lat,  lateral; mPFC,  medial prefrontal cortex; OFC,  orbitofrontal cortex; PCC,  posterior cingulate cortex; R,  right; Sub,  subgenual; TPJ,  temporo-parietal junction; VFC,  ventral frontal cortex.

Supplementary Figure 3. Scatter plot of functional connectivity in the region demonstrating baseline group differences between ADHD and control. Bars represent 1 SD.
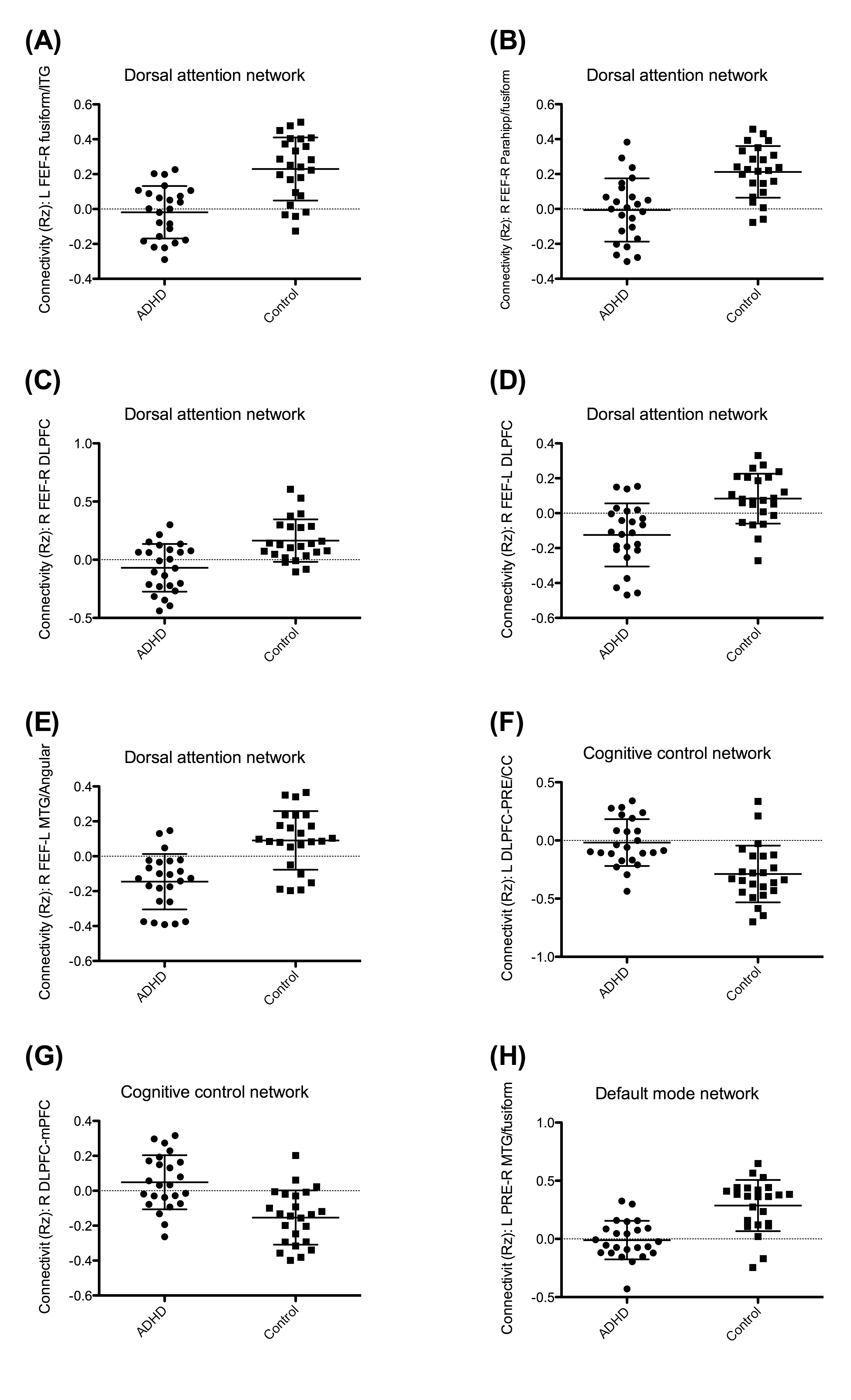


Supplementary Figure 4. Plot for time × treatment interaction (green line: atomoxetine-treated group, blue line: placebo-treated group).


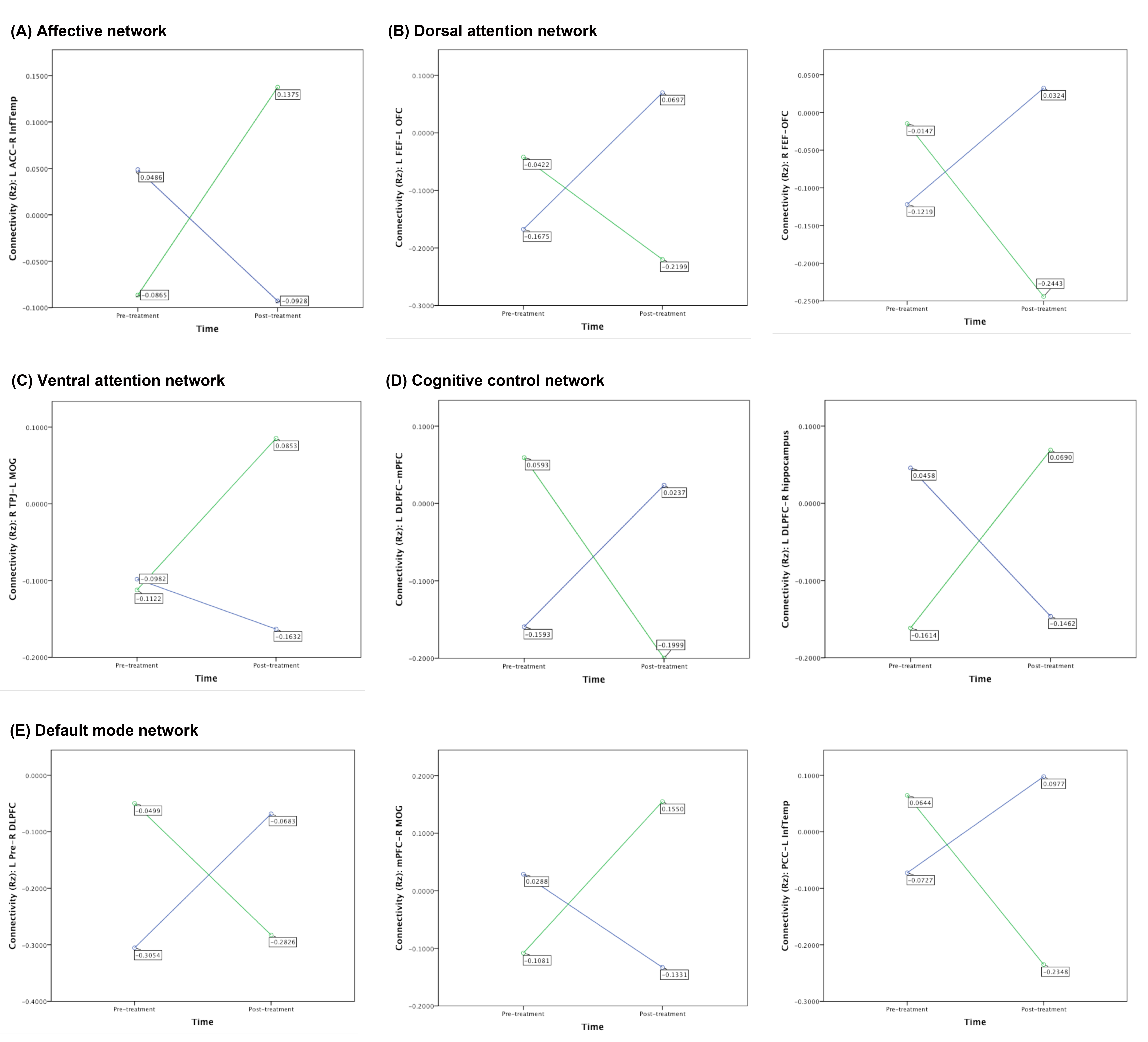

Supplement: supplementary Material [file pyv094SupplementaryIntro.docx]
